# Adaptation to bile and anaerobicity limits *Vibrio cholerae* phage adsorption

**DOI:** 10.1128/mbio.01985-23

**Published:** 2023-10-26

**Authors:** Zoe Netter, Drew T. Dunham, Kimberley D. Seed

**Affiliations:** 1 Department of Plant and Microbial Biology, University of California, Berkeley, Berkeley, California, USA; University of Pittsburgh, Pittsburgh, Pennsylvania, USA

**Keywords:** bacteriophages, phage defense, outer membrane, lipopolysaccharide

## Abstract

Bacteriophages (viruses of bacteria) play a pivotal role in shaping both the evolution and dynamics of bacterial populations. Bacteria employ arsenals of genetically encoded phage defense systems, but can alternatively achieve protection by changing the availability of cellular resources that phages rely on for propagation. These physiological changes are often adaptive responses to unique environmental signals. The facultative pathogen *Vibrio cholerae* adapts to both aquatic and intestinal environments with niche-specific physiological changes that ensure its evolutionary success in such disparate settings. In both niches, *V. cholerae* is susceptible to predation by lytic phages like ICP1. However, both phages and susceptible bacterial hosts coexist in nature, indicating that environmental cues may modulate *V. cholerae* cell state to protect against phage infection. This work explores one such modification in response to the intestine-specific signals of bile and anaerobicity. We found that *V. cholerae* grown in these conditions reduces O1-antigen decoration on its outer membrane lipopolysaccharide. Because the O1-antigen is an essential moiety for ICP1 phage infection, we investigated the effect of partial O1-antigen depletion as a mechanism of phage defense and observed that O1-depletion limits phage adsorption. We identified mechanistic contributions to O1-depletion, including the essentiality of a weak acid tolerance system for O1 production at low pH and alterations in transcriptional profiles indicating limitations in resources for O1-biosynthesis. This analysis illustrates a complex interplay between signals relevant to the intestinal environment and bacterial physiology that provides *V. cholerae* with protection from phage predation.

**IMPORTANCE:**

*Vibrio cholerae* is the bacterial pathogen responsible for cholera, a diarrheal disease that impacts people in areas without access to potable water. In regions that lack such infrastructure, cholera represents a large proportion of disease outbreaks. Bacteriophages (phages, viruses that infect bacteria) have recently been examined as potential therapeutic and prophylactic agents to treat and prevent bacterial disease outbreaks like cholera due to their specificity and stability. This work examines the interaction between *V. cholerae* and vibriophages in consideration for a cholera prophylaxis regimen (M. Yen, L. S. Cairns, and A. Camilli, Nat Commun 8:14187, 2017, https://doi.org/10.1038/ncomms14187) in the context of stimuli found in the intestinal environment. We discover that common signals in the intestinal environment induce cell surface modifications in *V. cholerae* that also restrict some phages from binding and initiating infection. These findings could impact considerations for the design of phage-based treatments, as phage infection appears to be limited by bacterial adaptations to the intestinal environment.

## INTRODUCTION

Bacteria thrive in spite of the constant threat of infection by predatory bacteriophages (or phages, viruses of bacteria). Selective pressure imposed by phage predation shapes bacterial populations and evolutionary trajectories (1). As much as 10% of a bacterial genome can be dedicated to phage defense (2), and phage defense genes can constitute major genomic differences contributing to inter- and intra-species diversity (3). There has been a recent acceleration in the discovery of diverse and innovative systems that defend bacteria from phage predation, from just a few simple genes providing defense like restriction-modification systems (4) to complex systems like CRISPR-Cas (5) and phage satellites (6, 7). However, a bacterium’s capacity to limit phage infection does not depend only on genetically encoded systems. Bacterial physiology also plays a pivotal role in phage defense. Global bacterial transcription and translation activities, metabolism, and availability of cellular components can impact the efficiency of phage infection, even in nearly identical host strains (8). While some phage defense systems have been shown to manipulate bacterial physiology to favor host population survival (such as a group of Sir2-domain-containing systems that respond to phage infection by degrading cellular NAD+, a necessary coenzyme in central carbon metabolism (9–11)), the impact of bacterial host physiology on phage infection is largely not mechanistically understood. This knowledge gap is compounded by the dynamic interplay between cell state and environment, which is fairly static in a laboratory setting but varies dramatically in a bacterium’s natural environment.

*Vibrio cholerae*, the bacterial agent that causes the diarrheal disease cholera, has widely variable transcriptional activity and metabolic states in its natural ecological niches. *V. cholerae* has evolved several intricate mechanisms to sense its environment and alter cellular state accordingly to adapt rapidly to disparate conditions. When the bacteria are ingested from an aquatic reservoir and enter a human host, they sense environmental cues like bile acids, anaerobicity, pH, and changes in osmolarity and temperature (12, 13). These intestinal stimuli trigger the *V. cholerae* ToxR regulon virulence cascade, which (via ToxT) activates the expression of cholera toxin (CTX), toxin co-regulated pilus (TCP), and other factors necessary to establish infection and support cholera pathogenesis (12). Late in infection, gene expression shifts to make global alterations in metabolism in preparation for persistence in the stool or aquatic reservoir (14), where a different set of genes appears necessary for survival (15). In addition to fluctuating environmental conditions, *V. cholerae* also encounters predation by lytic phages in both the human gut and the aquatic reservoir (16, 17). *V. cholerae* encodes a broad arsenal of anti-phage systems (7, 18, 19), but beyond a few genetically encoded means to restrict phage infection, little is known about the influence of pertinent intestinal stimuli on *V. cholerae’s* susceptibility to phage predation. There is evidence that genetically “defenseless” strains can be co-isolated with lytic phages from cholera patient stool samples, indicating coexistence in the human gut (18). However, it is unclear whether this is due to spatial separation resulting from the heterogeneity of the gut environment and/or by changes in bacterial physiology and metabolism in the gut environment that also impact phage susceptibility.

The bacterial cell surface provides the first line of defense against phage attack and environmental challenge. In Gram-negative bacteria like *V. cholerae*, the surface outer membrane is composed of a phospholipid bilayer embedded with lipopolysaccharide (LPS, composed of a bilayer-embedded lipid A component, a core-polysaccharide linker, and a widely variable O-antigen polysaccharide component) in the outer leaflet along with proteins involved in transmembrane transport and environmental sensing (20). These outer membrane components are frequently recognized by phages as receptors during the attachment process (21, 22). Phage receptor display can be modified directly by changes in the expression of their corresponding biosynthetic genes or by other changes in metabolism that alter the availability of the components necessary for biosynthesis or transport (20). Although over 200 *V*. *cholerae* O-antigen serogroups have been identified, the vast majority of cholera infections are caused by the O1 serogroup (23). The O1-antigen component of *V. cholerae* LPS is also the receptor for the dominant lytic vibriophage ICP1, which is recurrently co-isolated with *V. cholerae* in patient stool samples in areas where cholera is endemic (24). *V. cholerae* is heavily restricted from permanently losing the LPS O1-antigen to escape ICP1 predation because it is required for efficient colonization of the intestine and subsequent pathogenesis (25).

The coexistence of susceptible *V. cholerae* and ICP1 phages has been noted in several instances in cholera patient stool samples (16, 17, 26, 27), leading us to hypothesize that some aspect of the human intestinal environment may alter phage susceptibility. Seeking to determine the physiological impact of specific gut-associated stimuli on *V. cholerae* and the repercussions for ICP1 infection, we focused specifically on bile acids and anaerobicity. Both of these stimuli are known to cause broad transcriptional changes in *V. cholerae* (13). We assess the individual and combinatorial effects on *V. cholerae* LPS production and identify a combination of anaerobicity and bile acid supplementation that reduces the degree of O1-decoration of LPS in *V. cholerae*. We utilize phage-based assays to assess the biological relevance of this phenomenon and transcriptomics to identify several potential mechanisms for decreased O1-antigen production, including widespread transcriptional changes to central metabolic processes, reduced production of O1 biosynthetic enzymes, and the essentiality of weak acid tolerance for O1-antigen production at low pH.

## RESULTS

### *Vibrio cholerae* dynamically modifies the abundance of O1-decorated LPS in response to intestine-derived stimuli

**Fig 1:**
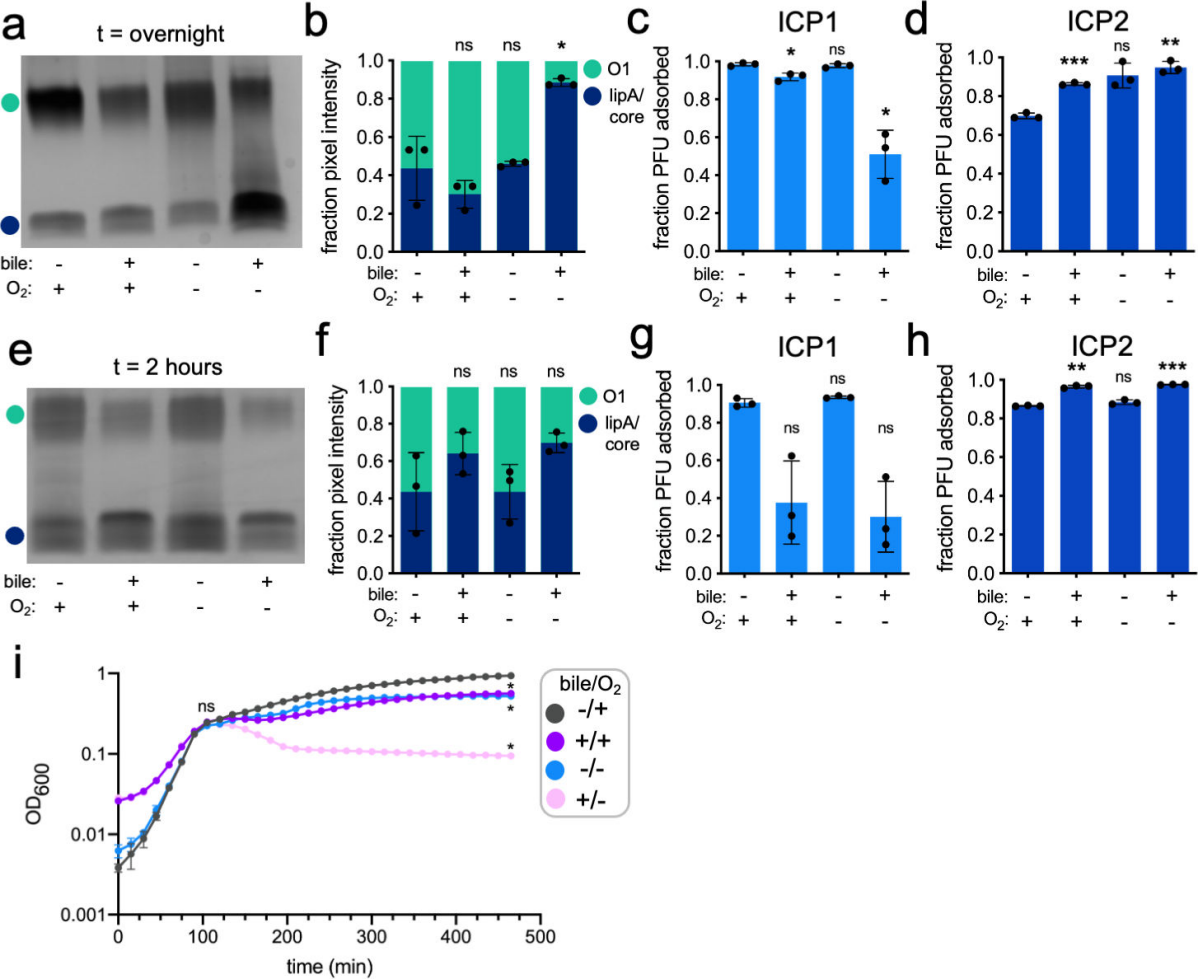
*Vibrio cholerae* dynamically modifies the abundance of O1-decorated LPS in response to intestine-derived stimuli (a) Silver stain of LPS purified from *V. cholerae* cultured overnight in LB or LB supplemented with 0.5% bile acids (bile ±), aerobically or anaerobically (O_2_ ±). (b) Average fraction of pixel intensity quantified from replicate purified LPS silver stains. Light green represents the relative fraction of O1-antigen signal, and dark blue represents the fraction of lipid A/core signal. *P = 0.0417 by ANOVA followed by Welch’s *t*-tests comparing the mean in each non-standard condition to aerobic LB, ns = nonsignificant. (c) Fraction of O1-dependent phage ICP1 adsorbed by *V. cholerae* cultured overnight in all combinations of culture conditions. *P < 0.05 by ANOVA followed by Dunnett’s T3 multiple comparisons test using aerobic LB as the control condition, ns = nonsignificant. (d) Fraction of OmpU-dependent phage ICP2 adsorbed by *V. cholerae* cultured overnight in all combinations of culture conditions. **P = 0.0026 and ***P = 0.001 by ANOVA followed by Dunnett’s T3 multiple comparisons test using aerobic LB as the control condition, ns = nonsignificant. (e–h) Same as (a–d) with culture harvested at 2 hours. For (h), **P = 0.0037 and ***P < 0.0001 by ANOVA followed by Dunnett’s T3 multiple comparisons test using aerobic LB as the control condition, ns = nonsignificant. (i) Growth curve measuring OD_600_ for all combinations of culture conditions over time (in minutes). *P < 0.0001 by ANOVA followed by Dunnett’s T3 multiple comparisons test using aerobic LB as the control condition at the final timepoint.

To examine the effect of gut-specific cues on *V. cholerae* LPS, we cultured *V. cholerae* overnight in LB media supplemented with 0.5% bile both aerobically and anaerobically and then extracted and visualized LPS by denaturing gel electrophoresis and silver stain. *V. cholerae* cultured with bile supplementation alone or anaerobicity alone produced LPS similar to cells in standard LB aerobic culture, while *V. cholerae* grown in a combination of bile and anaerobic conditions produced LPS with strikingly less O1-decoration than the other conditions (Fig. 1a). While the reduction in O1-antigen was visually apparent, it was not a complete depletion (~50% less average O1 signal intensity) (Fig. 1b).

We were curious to assess if the degree of reduction in O1-decorated LPS observed under anaerobic conditions with bile was biologically consequential for phage infection, specifically for the O1-dependent phage ICP1. We probed phage susceptibility in these different conditions with a modified adsorption assay, heat-killing cells grown in each condition to control for differences in host cell physiological state. The physiological state of host cells can dramatically alter the robustness of the phage life cycle after initial genome injection (28), but measuring the capability of ICP1 to adsorb heat-killed cells bypasses the challenges of comparing phage replication in hosts with different metabolic states and allows us to directly assess phage receptor availability. ICP1 efficiently adsorbed to *V. cholerae* cells cultured in standard, anaerobic, and aerobic bile conditions; however, ICP1 adsorption was reduced by ~50% when cells were cultured anaerobically with bile (Fig. 1c). Although the O1-antigen was not completely eliminated from LPS on cells in this condition, this indicates that the observed reduction in O1-decorated LPS has a sizeable impact on ICP1 adsorption. To further validate that the modified adsorption assay faithfully measures phage receptor availability, we repeated the experiment using two unrelated vibriophages: ICP2, a podovirus that uses the outer membrane porin OmpU as its receptor (29), and ICP3, an O1-dependent podovirus (Fig. S1A). Expression of the ICP2 receptor *ompU* is known to be upregulated in bile conditions (12, 30). Concordantly, we observed enhanced ICP2 adsorption in bile-supplemented conditions (Fig. 1d). Comparable to ICP1, we observed a similar defect in ICP3 adsorption only with cells cultured overnight in the anaerobic bile condition (Fig. S1B). These results validate that the reduction in O1-antigen abundance observed by purified LPS silver stain broadly impacts *V. cholerae* susceptibility to O1-dependent phages like ICP1 and ICP3, suggesting that such adaptations could affect *V. cholerae*’s susceptibility to phages during intestinal colonization.

Reduction in O1-decorated LPS can result from genetic mutations in the O1-antigen biosynthetic gene cluster (24). If overnight growth in the anaerobic bile culture condition selects for mutants that produce less O1-antigen, then the reduced O1 phenotype from growth overnight in this condition would be expected to persist with outgrowth in the absence of such stimuli (i.e., in standard aerobic LB). In this case, O1 mutants would also be expected to have increased fitness in anaerobic bile conditions. Conversely, if the reduction in O1-decorated LPS is a result of transient phenotypic changes rather than permanent genetic changes, cultures grown overnight in anaerobic bile conditions would be expected to recover complete O1-antigen production when switched to standard conditions. To distinguish between these possibilities, we tested the recovery of anaerobic bile overnight *V. cholerae* cultures in standard aerobic conditions and observed complete recovery of O1-decorated LPS within 120 min (Fig. S2A through E). We also tested the growth efficiency of a genetic mutant (Δ*wbeL*) that produces no O1-antigen and found no difference in fitness relative to wild-type *V. cholerae* in either aerobic or anaerobic conditions with varying concentrations of bile (Fig. S2F and G). We were also curious if this response was conserved among *V. cholerae* strains. We grew both classical *V. cholerae* and contemporary El Tor clinical isolates in anaerobic bile conditions and observed reductions in O1-antigen production similar to the *V. cholerae* E7946 strain used as our laboratory strain (which is also an El Tor clinical isolate) (Fig. S11), indicating that O1-antigen reduction is likely conserved across the serogroup. Taken together, these data suggest that the observed reduction in O1-decorated LPS produced by *V. cholerae* in anaerobic bile conditions is due to a reversible alteration in pathway(s) involved in O1-antigen biosynthesis.

LPS molecules are built from organic components of central carbon metabolism (the O1-antigen is synthesized from malate and D-fructose 6-phosphate) (31). LPS biosynthesis may be impacted by the metabolic state of the cell simply due to different resource availability in different conditions. More stressful culture conditions could cause diminished production of central carbon metabolism components in order to decrease cell growth, as has been demonstrated in a dual transcriptomic and metabolomic analysis of the *E. coli* stress response (32). Depletion of central carbon metabolism intermediates would effectively deplete the pool available for synthesizing the O1-antigen component of LPS, resulting in incomplete O1-decoration on the cell surface. We observed the depletion of O1-decorated LPS in overnight cultures grown anaerobically with bile, a condition where cells may be actively altering their metabolic states to ensure continued survival. To evaluate cell growth in all relevant conditions, we measured *V. cholerae* growth in each condition over time (Fig. 1i). All cultures grew similarly for the first ~120 min; however, as the rest of the cultures proceeded to grow, the cells in bile anaerobic conditions instead persisted at a low but stable OD and CFU (Fig. 1i; Fig. S10A). These data illustrate that while the end state of cells in each culture condition may vary dramatically, there are earlier instances where growth rates were more similar.

We were interested in assessing the state of cell surface LPS at the 2-hour timepoint, where although the metabolic states of the cells are likely still fundamentally different, the relative growth rates appear similar regardless of culture condition. At 2 hours, we were surprised to observe a slight, though nonsignificant, reduction in O1-decorated LPS in the presence of bile under both aerobic and anaerobic conditions. The difference was subtle by quantification of purified LPS silver stain (Fig. 1e and f) and more variable but not significantly different when measured by ICP1 adsorption assay (Fig. 1g). ICP2 and ICP3 adsorption remained fairly robust and consistent across all conditions at 2 hours (Fig. 1h; Fig. S1C). This suggested that at the 2-hour timepoint, there are unexplainable differences, perhaps related to the accessibility of different portions of the O1-antigen on the cell surface that may cause variability in ICP1 adsorption. However, the O1-antigen appears to be the major determinant of adsorption, as ICP3 adsorption at this timepoint was the same across conditions. While the specific differences in *V. cholerae* cell surface composition in overnight compared to exponential phase are unknown, Gram-negative bacteria in the stationary phase (overnight culture) generally tend to be smaller than those in the exponential phase (2-hour culture), with major differences in membrane composition that alter rigidity and permeability (33). The state of the membrane in the exponential growth phase could impact the relative adsorption efficiency of ICP1 and ICP3, contributing to the variability of ICP1 adsorption at the 2-hour timepoint. Together, these results implicated a dynamic cellular response to bile early in the growth of the culture and anaerobic bile in a subsequent overnight culture that negatively impacted *V. cholerae* O1-antigen production. The combination of bile and anaerobicity in overnight culture maintains low levels of O1-antigen production, and O1-antigen reduction negatively impacts ICP1 adsorption in all instances.

### RNA sequencing of *V. cholerae* grown in anaerobic, bile, and anaerobic bile conditions reveals transcriptional alterations in global cellular processes and pathogenesis genes

In order to identify transcriptomic changes underlying the observed reduction in O1-decorated LPS, we conducted RNA sequencing for all four culture conditions at both 2-hour and overnight timepoints. We examined all pairwise comparisons of conditions where the O1-antigen appeared to be depleted in the experimental condition but not in the control condition (subtle nonsignificant differences at the 2-hour timepoint: aerobic bile vs aerobic and anaerobic LB, and anaerobic bile vs aerobic and anaerobic LB; significant differences at overnight timepoint: anaerobic bile vs all other conditions), reasoning that any overlap of the differentially expressed genes between conditions may identify candidates involved in the observed depletion of the O1-antigen. Only eight genes were consistently differentially expressed across all seven pairwise comparisons from both timepoints (see Text S1). In all conditions comparing a bile-supplemented culture to a non-supplemented LB culture, we observed upregulation of genes involved in bile RND efflux (resistance-nodulation-division efflux superfamily) *(vexABCD* (34)), indicating that our transcriptomics experiment successfully recapitulated a known transcriptional response to bile in *V. cholerae* (35) (Fig. S3D, S4D and E). The general lack of overlap between the 2-hour and overnight data sets further indicated that the subtle variation in O1-antigen observed at 2 hours was likely not significant and not driven by a distinguishable transcriptional response shared with the overnight timepoint. Hence, we focused our follow-up analysis primarily on the overnight timepoint. Additionally, none of the differentially expressed genes shared across all comparisons had direct logical connections to O1-antigen biosynthesis, indicating that instead of a simple conserved transcriptional mechanism, multiple factors may be involved in responses to both bile and anaerobicity that collectively impact O1-production.

**Fig 2:**
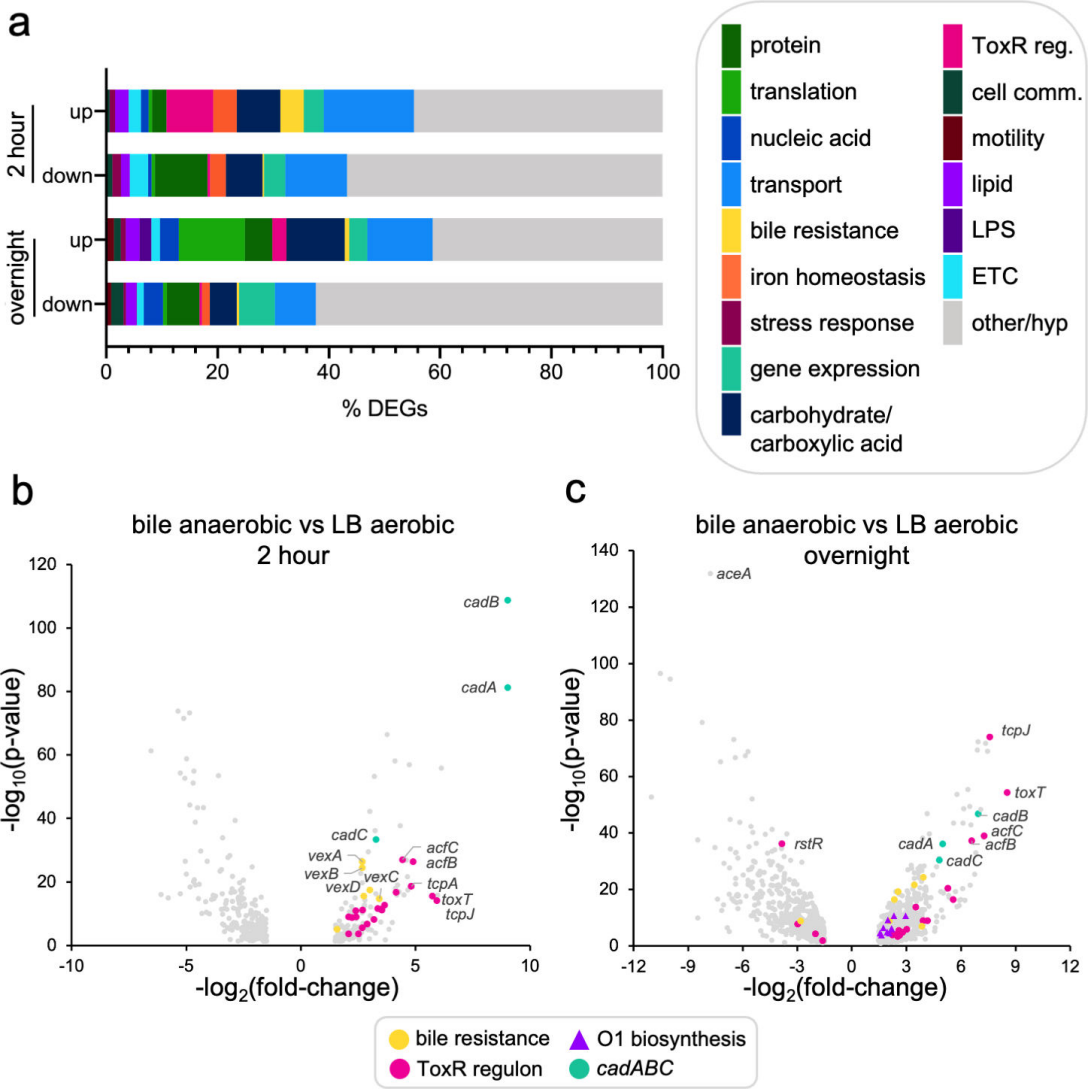
RNA sequencing of *V. cholerae* grown in anaerobic, bile, and anaerobic bile conditions reveals transcriptional alterations in global cellular processes and pathogenesis genes. (a) Stacked bar graph representing the proportion of differentially expressed genes (DEGs) representing each considered gene ontology category. Differentially expressed genes from each individual comparison are pooled in this analysis. Categories represent gene ontology metabolic processes unless otherwise denoted. “ToxR reg.,” ToxR regulon; “cell comm,” cell communication; and “hyp,’” hypothetical. (b) Two-hour and (c) overnight volcano plots of differentially expressed genes in anaerobic bile compared to aerobic LB conditions. Adjusted *P*-value ≤ 0.05 and fold change ≥ ±1.5 were considered significant, and plotted, nonsignificant results are excluded from the plot. Select gene categories of interest are highlighted in color and indicated in the legend. See Fig. S3 and S4 for volcano plots with designated gene ontology categories.

Given the lack of clear candidate genes responsible for decreased O1-antigen levels, we used gene ontology to predict what metabolic processes were represented in the differentially expressed genes from each comparison. Gene ontology analysis revealed that genes encoding products involved in transmembrane transport dominated the pool of differentially expressed genes in O1-deplete conditions, both up- and downregulated (Fig. 2a; Fig. S3 and S4). More noticeably at the overnight timepoint, upregulated genes in O1-deplete conditions tended to be enriched in functions related to protein synthesis, e.g., translation, ribosome biogenesis, amino acid metabolism, and tRNAs, while the downregulated set was slightly enriched for genes involved in the regulation of gene expression and other/unclassified genes (Fig. 2a; Fig. S3 and S4). The transcriptomic data are consistent with a cell state where there may be adequate resources for transcriptional upregulation but limited resources to facilitate protein synthesis. Together, these data indicate that our transcriptomic profiling captured both previously described and novel transcriptional adaptations to our tested conditions (see Text S1).

Many upregulated genes with the highest fold changes in all pairwise comparisons were components of the ToxR regulon, specifically *toxT* (the transcriptional activator of downstream virulence cassettes CTX and TCP) (36), the majority of TCP components (37), and accessory colonization factors *(acfA* (38), *acfB* (39), *acfC* (40)) (Fig. 2b and c; Fig. S5). We observed the activation of *ctxAB(*41) and concordant downregulation of *rstR* (42), the negative regulator of CTX, at the 2-hour timepoint (Fig. S5B). Also indicative of ToxR regulon activity and bile resistance, *ompU* expression was increased in overnight comparisons, although the corresponding downregulation of *ompT* (30) was only observed in one of the overnight comparisons (Fig. S5B). Bile and anaerobicity are both known activators of the ToxR regulon (43, 44), which is consistent with the observation of its transcriptional upregulation in these conditions. ToxR induces a regulatory cascade for *V. cholerae* virulence gene expression induced in the human gut in the context of infection (12) and has been shown to be highly transcriptionally upregulated in both rabbit and mouse animal models of cholera (45). Interestingly, several other features of the distinct transcriptional profiles of *V. cholerae* in rabbit and mouse infections are also represented in the 2-hour bile and overnight anaerobic bile culture conditions. This includes activation of a colonization factor (*vc1773*) (45) encoded in *Vibrio* pathogenicity island 2 (VPI-2) and genes involved in the utilization of host-derived long-chain fatty acids found in cecal fluid (e.g., long-chain fatty acid transport, acetyl CoA dehydrogenases, and glycerol transport and metabolism genes, which enable utilization of fatty acids) (45) (Fig. S3 and S4; Data Sheet 3). At the overnight timepoint, we also observed upregulation of *almEFG* lipid A modification genes which are thought to be important in providing protection against antimicrobial peptides found in the gut (46, 47) (Fig. S6). Taken together, the data sets suggest that *V. cholerae* producing less O1-decorated LPS in these culture conditions have also made global shifts in gene expression to a state remarkably similar to what has been observed in animal models of *V. cholerae* infection.

### Bile and anaerobicity alter the transcription of a subset of genes in the O1-biosynthetic cluster

**Fig 3:**
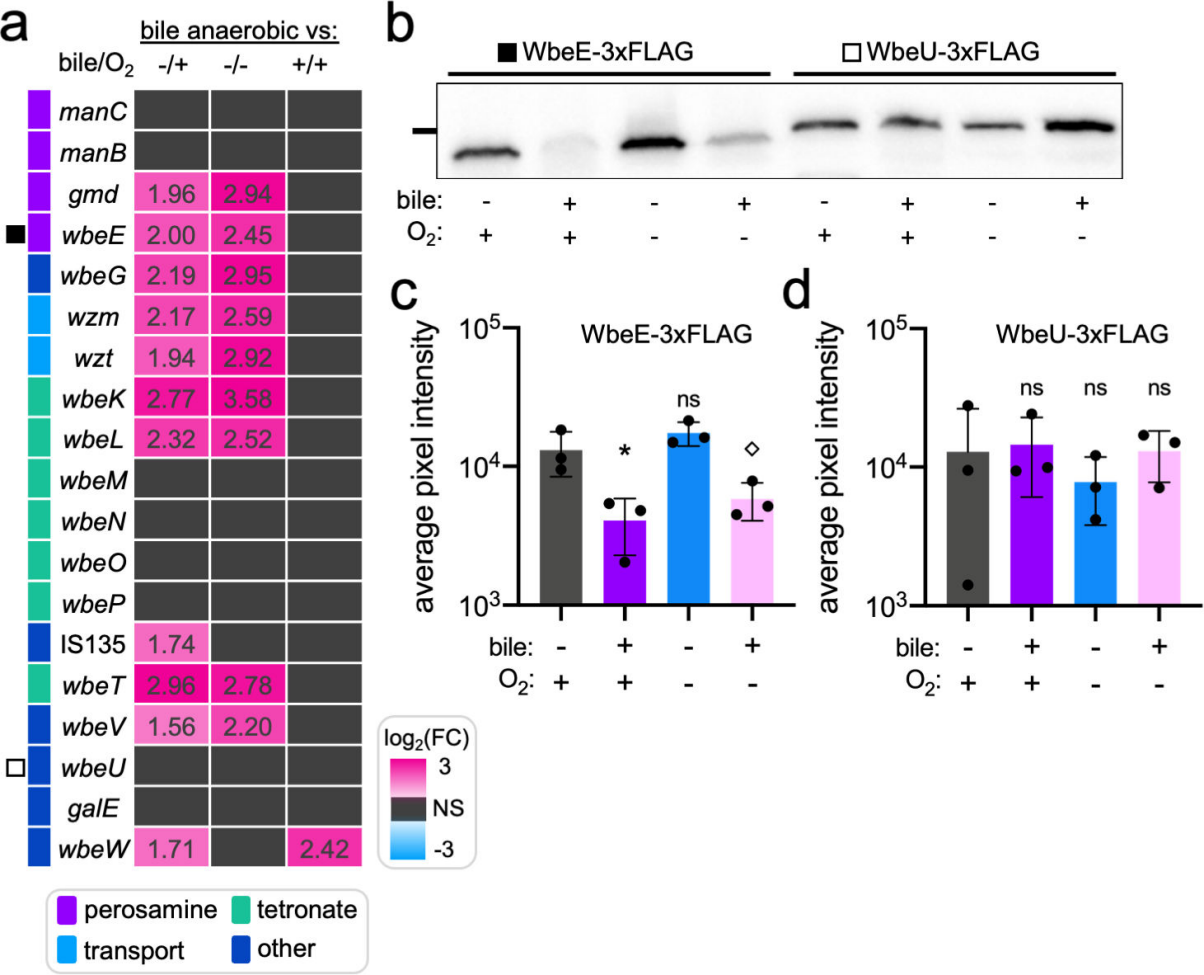
Bile and anaerobicity alter the transcription of a subset of genes in the O1-biosynthetic cluster. (a) Heatmap of log_2_(fold change) for genes in the O1-biosynthetic cluster in overnight culture comparisons. log_2_(fold change) values are listed in individual cells. Right legend gives colorimetric approximation of log_2_(fold change), NS (black) denotes nonsignificant changes in transcript levels (*P*_adj_ > 0.05). Bottom legend provides the function category for each gene (colored bars left of gene names). (b) Representative Western blot against FLAG-tag monitoring WbeE-3xFLAG (black square) and WbeU-3xFLAG (white square) protein expression in all combinations of culture conditions after overnight incubation. Left line represents 37 kDa marker. (c) WbeE and (d) WbeU pixel intensity quantification for replicating Western blot experiments. *P = 0.0204, diamond *P* = 0.0549 by ANOVA followed by Dunnett’s T3 multiple comparisons test using aerobic LB as the control condition, ns = nonsignificant.

We examined the O1-biosynthetic cluster itself to identify connections between growth conditions and the transcription of enzymes necessary to produce O1-antigen. To our surprise, seven consecutive genes within the O1-biosynthetic cluster *(gmd-wbeL* (48)) were significantly upregulated when comparing overnight anaerobic bile conditions to either aerobic or anaerobic LB (Fig. 3a). This locus contains genes required for the biosynthesis of both O1-antigen precursor components, perosamine and tetronate, as well as the required transport genes for O1-antigen assembly (48). We were not expecting to observe a corollary transcriptional upregulation in this genomic region in conditions where less O1-decorated LPS was produced, and so, we proceeded to investigate if the observed transcriptional upregulation resulted in increased protein production. A representative gene from within (*wbeE (*48)) and outside (*wbeU (*48)) the upregulated set of genes was tagged with a C-terminal FLAG tag, and protein production was monitored by Western blot after overnight culture. While WbeU production remained similar across all culture conditions (consistent with no significant changes in expression detected by RNA-seq), there was a striking reduction in the amount of WbeE detected in cultures containing bile (Fig. 3b through d). This suggested that the transcriptional upregulation within the O1-biosynthetic cluster was not indicative of enhanced protein production, but instead possibly indicated that cells were not producing enough of these enzymes and attempting to upregulate transcription to bolster low protein levels. Importantly, low abundance of WbeE was not sufficient to reduce O1-decorated LPS production, as similar levels of WbeE were produced in anaerobic and aerobic bile conditions, and we observed complete O1 production in aerobic bile overnight culture. Taken with the RNA-seq data suggesting a global upregulation of genes encoding ribosomal and other translational components (Fig. 2a; Fig. S3 and S4), these results suggest that cells in anaerobic bile conditions may be adjusting to conditions with fewer resources for protein production by enhancing transcription as if to prepare for the introduction of more protein synthesis resources. These global changes alone could result in less O1-decorated LPS production via a reduction in the pool of enzymes essential for O1 biosynthesis. This would represent a mechanism of phage resistance tied to alterations in cellular processes that result in the restriction of ICP1 phage predation via the reduced presentation of the phage receptor.

### The *cadABC* weak acid tolerance system is necessary for O1 biosynthesis in anaerobic bile conditions

**Fig 4:**
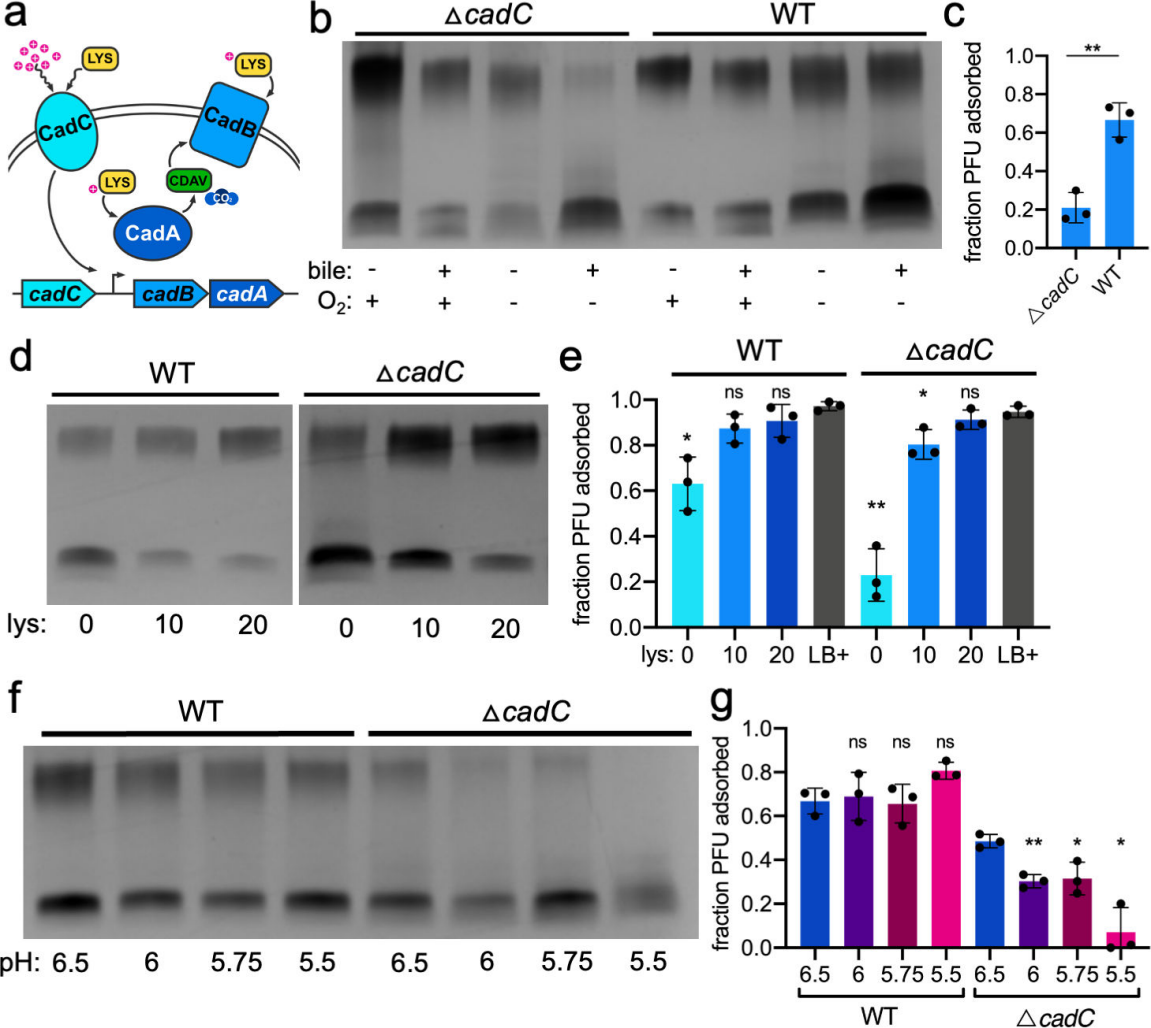
The *cadABC* weak acid tolerance system is necessary for O1 biosynthesis in anaerobic bile conditions. (a) Schematic of the *cadABC* weak acid tolerance system. At neutral pH, membrane-bound CadC represses the expression of *cadAB* (excluded from schematic). When CadC senses low extracellular pH and lysine, it activates the expression of *cadB* (a lysine/cadaverine antiporter) and *cadA* (a lysine decarboxylase that consumes protons [magenta circles with (+)] to convert lysine (LYS, yellow) to cadaverine (CDAV, green) and carbon dioxide. CadABC activity neutralizes intra- and extracellular pH. (b) Purified LPS silver stain of wild-type (WT) and Δ*cadC V. cholerae* in all combinations of culture conditions. (c) Fraction of ICP1 adsorbed to *V. cholerae* grown overnight in anaerobic bile culture conditions. **P = 0.0027 by Student’s *t*-test. (d) Purified LPS silver stain of *V. cholerae* grown overnight in anaerobic bile culture conditions supplemented with L-lysine (lys, concentration denoted in mM). (e) Fraction of ICP1 adsorbed to *V. cholerae* grown overnight in anaerobic bile culture conditions supplemented with L-lysine (lys, mM) or aerobic LB (LB+). *P < 0.05, **P = 0.0065 by ANOVA followed by Welch’s *t*-tests comparing the mean in each lysine-supplemented condition to aerobic LB, ns = nonsignificant. (f) Purified LPS silver stain of wild-type (WT) and Δ*cadC V. cholerae* grown overnight in anaerobic bile culture conditions, pH reduced with malic acid. (g) Fraction of ICP1 adsorbed to *V. cholerae* grown overnight in anaerobic bile culture conditions, pH reduced with malic acid. *P < 0.05 and **P = 0.0018 by ANOVA followed by Welch’s *t*-tests comparing the mean in each reduced-pH condition to pH 6.5, ns = nonsignificant.

From the pool of differentially expressed genes identified by RNA-seq, we selected and screened several candidate genes and systems with potential relevance to O1-antigen depletion in bile anaerobic conditions. We focused on genes implicated in bile resistance and bile-mediated transcriptional response [*vexABR* (49), *leuO* (50)*, toxR* (51)] and systems with predicted utility in intestinal colonization (PTS carbohydrate transport system (52), RTX toxin gene cluster (53), *almEFG* lipid modification system (46), citrate utilization, *cadABC* weak acid tolerance system (54, 55), and disabled a system’s regulator whenever possible. Surprisingly, these systems were all dispensable for growth in anaerobic bile conditions, and nearly all mutants were able to synthesize as much O1-decorated LPS as wild-type *V. cholerae* (Fig. S11). A single exception was the Δ*cadC* mutant lacking the CadC sensor/transcriptional activator protein of a lysine-dependent weak acid tolerance system (Fig. 4a). The Δ*cadC* strain produced LPS with even less O1-antigen decoration than wild-type *V. cholerae* in anaerobic bile conditions and concordantly demonstrated a further reduction in ICP1 adsorption (Fig. 4b and c; Fig. S7A). In response to low pH and lysine, CadC activates the expression of *cadA* (a lysine decarboxylase that consumes protons and converts lysine to cadaverine) and *cadB* (a lysine/cadaverine antiporter) (54, 55) (Fig. 4a). RNA-seq data also implicated the importance of this gene cluster, as *cadB* and *cadA* were among the most significantly differentially expressed genes in both the 2-hour and overnight timepoint comparisons (Fig. 2b and c; Fig. S4). To confirm that the activity of the *cadABC* system was required for maximum O1-antigen biosynthesis, we cultured a Δ*cadA* strain in anaerobic bile conditions and observed a decrease in O1-decorated LPS production on par with the Δ*cadC* strain (Fig. S8C and D). A further reduction in O1-antigen with loss of the weak acid tolerance system activity indicated that the *cadABC* system is functionally necessary for O1-decorated LPS production in anaerobic bile conditions.

While the loss of weak acid tolerance further depleted the production of O1-decorated LPS, none of the screened mutants were able to produce more O1-decorated LPS than wild-type *V. cholerae* in anaerobic bile conditions (Fig. S11). We tested the chromosomal overexpression of *cadC* by synthetic induction in anaerobic bile conditions and did not observe any increase in O1-decorated LPS (Fig. S7B, S8A and B). This could be indicative of limitations in cellular resources necessary for O1-antigen biosynthesis that are not replenished by the de-acidification activity of *cadABC*. Given that cells may lack robust protein synthesis capacity, we do not have direct evidence that synthetic overexpression can increase CadC protein concentration in these conditions. Taken together, these results suggest that *cadABC* activity is necessary to produce O1-decorated LPS in anaerobic bile conditions, but the reduction of O1-decorated LPS in wild-type cells may not be due to a simple lack of adequate CadABC system activity and could instead be a result of other metabolic changes interfering with cellular resources necessary for weak acid tolerance and O1 biosynthesis. Therefore, weak acid tolerance is also necessary for ICP1 infection in anaerobic bile conditions, as its activity is required for the production of the phage receptor.

### pH determines the degree of O1-decorated LPS production

Because synthetic overexpression of *cadC* did not result in the recovery of O1-decorated LPS, we sought to alternatively activate the *cadABC* system with the addition of exogenous lysine, a trigger of CadC activation. Initially, we were excited to observe that the addition of lysine appeared to have a positive, dose-dependent effect on O1-decorated LPS production in wild-type *V. cholerae*, with 20 mM lysine supplementation of anaerobic bile *V. cholerae* cultures resulting in LPS comparable to what is observed in aerobic LB conditions (Fig. 4d and e; Fig. S7G). To determine if the recovery of O1-decorated LPS was due to the activation of *cadABC*, we grew the Δ*cadC* strain with supplemental lysine, anticipating that lysine would no longer impact the production of O1-decorated LPS. Unexpectedly, however, we observed the recovery of O1-decorated LPS with lysine supplementation for the Δ*cadC* mutant comparable to wild-type *V. cholerae* (Fig. 4d and e; Fig. S7G). The Δ*cadA* strain also recovered production of O1-decorated LPS in anaerobic bile following supplementation with lysine (Fig. S8C, D and S7G). While the addition of lysine restored the production of O1-decorated LPS and correspondingly restored sensitivity to ICP1 adsorption (Fig. 4e), bacterial growth did not increase (Fig. S10E through G). This indicates that general growth restriction is likely not the cause of O1-decoration loss and reduced phage adsorption in anaerobic bile conditions. Overall, these results suggested that the recovery of O1-antigen was not directly due to lysine-mediated activation of *cadABC*.

Lysine is a basic amino acid and *cadABC* is responsive to acidic conditions, so we considered the possibility that the addition of lysine was increasing the media pH, and this alkalization was the underlying cause of O1-antigen recovery during lysine supplementation. We first measured the pH of fresh and spent media from each relevant condition and confirmed that lysine supplementation increases pH from 6.8 to ~8.5 in anaerobic bile culture (Fig. S8E). We therefore supplemented anaerobic bile cultures with arginine (another basic amino acid) at the same concentrations or adjusted the pH of the culture media with sodium hydroxide to match 10 mM (~pH 7.5) and 20 mM (~pH 8.5) basic amino acid supplementations and assessed the resulting LPS. Both arginine and sodium hydroxide supported robust recovery of O1-decorated LPS in both wild-type and Δ*cadC V. cholerae* (Fig. S8F and G), indicating that increased pH allows for complete O1-antigen production.

The pH of anaerobic bile media (both fresh and spent) was very close to neutral (~pH 6.8) (Fig. S8E), while in the context of human infection, *V. cholerae* experiences dramatic pH ranges in bile (~pH 6–8) (56) and stomach acid (~pH 2–6) (57). Because of the apparent essentiality of *cadABC* weak acid tolerance activity for O1 biosynthesis in anaerobic bile conditions, we hypothesized that reducing the pH in this condition could necessitate further increased CadAB activity for O1-production, meaning wild-type *V. cholerae* with active CadAB may continue to synthesize O1 until the pH is too low to be tolerated, while mutants in the weak acid tolerance system would lose O1-biosynthetic capacity at sufficiently low pH. We therefore examined the effects of low pH anaerobic bile conditions on LPS production in both wild-type *V. cholerae* and the Δ*cadC* strain. The wild-type strain was able to grow in bile anaerobic conditions as acidic as pH 5.5 (Fig. S10H) and did not exhibit any significant changes in O1-decorated LPS production at low pH (Fig. 4f and g). While the Δ*cadC* strain grew at relatively similar rates as the wild-type at low pH (Fig. S10H), the LPS produced by this strain exhibited a further reduction in O1-antigen substitution with decreasing pH, where almost no detectable O1-antigen was produced at pH 5.5 (Fig. 4f and g). We used an organic acid (malic acid) to reduce the pH of the media; however, *cadABC* has been previously described as important for tolerance to both organic and inorganic acids in *V. cholerae* (58). Accordingly, when we reduced the pH of the media with an inorganic acid (hydrochloric acid), we observed the same dose-dependent reduction in O1-antigen in the absence of *cadABC* activity (Fig. S9A, B and S10I), suggesting that the phenotype is truly pH dependent and independent of the origin of protons causing the acidity.

Because the acidity of the anaerobic bile condition appeared to be the determining factor in reducing O1-antigen production, we sought to determine if acidity was sufficient to reduce O1-decoration on LPS, or if bile supplementation and/or anaerobicity were also required. We measured growth and LPS production at low pH in anaerobic LB (Fig. S10J, S8C and D) and aerobic bile conditions (Fig. S10K, S9E and F) and did not observe any reduction in the degree of O1-decorated LPS produced by either wild-type or Δ*cadC* strains. These results suggest that bile, anaerobicity, and low pH combined reduce the production of O1-decorated LPS.

Lastly, we utilized the observations that high pH and weak acid tolerance modulate O1-antigen production in anaerobic bile conditions to assess whether a reduction in phage adsorption protects *V. cholerae* populations from phage infection. We infected overnight anaerobic bile cultures with ICP1 to directly compare *V. cholerae* cell survival while modulating the degree of O1-decoration. We observed ~15% survival of the wild-type population after a single round of ICP1 infection in anaerobic bile conditions, while the *cadC* mutant that makes even less O1-antigen experienced enhanced protection from phage predation (Fig. S8H). Both wild-type and *cadC* mutant populations became completely susceptible to phage predation when the anaerobic bile media pH was increased to 8.5 (Fig. S8H), indicating that adsorption defects manifest as population protection from phage predation. Acid tolerance mediated by *cadABC* allows for partial O1-antigen production in anaerobic bile conditions, but the loss is significant enough to dramatically hinder the adsorption of ICP1 phage and enhance population survival, providing an environmentally mediated mechanism of adaptive phage resistance.

## DISCUSSION

**Fig 5:**
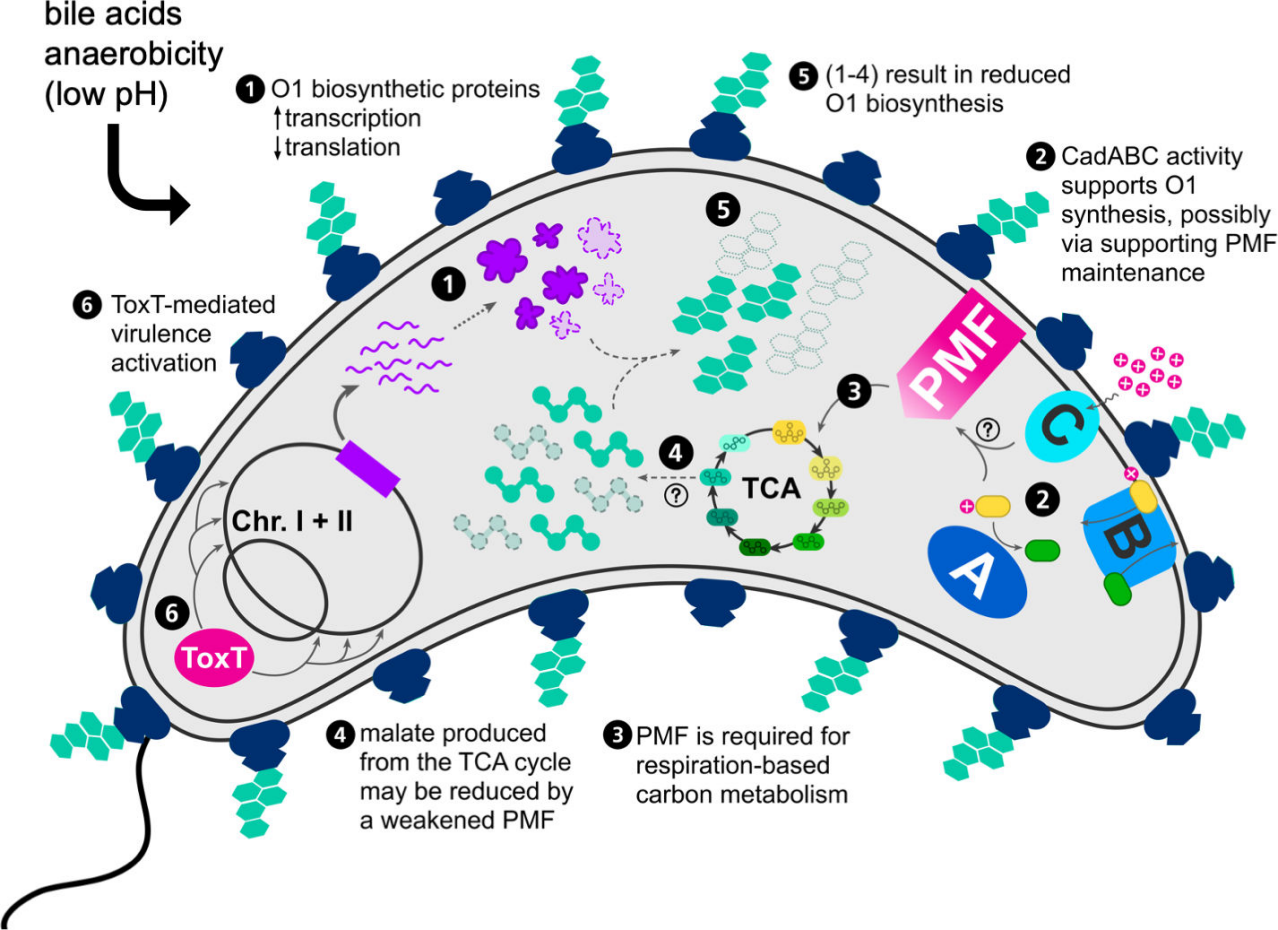
Mechanisms contributing to O1-antigen depletion in response to intestinal stimuli. (1) In O1-deplete conditions, pools of enzymes available for O1-antigen biosynthesis are reduced. This reduction is correlated with the transcriptional upregulation of a putative operon within the O1-biosynthetic cluster (Fig. 3). (2) The *cadABC* weak acid tolerance system is necessary for O1-antigen production in O1-deplete conditions (Fig. 4). CadABC activity neutralizes cellular pH and may play a role in the maintenance of proton motive force (PMF) in O1-deplete conditions, making its activity conditionally essential for robust carbon metabolism and O1 biosynthesis by proxy. (3) Cellular proton motive force (PMF) drives respiratory central carbon metabolism and ATP production. The tricarboxylic acid cycle (TCA) transfers electrons to cofactors (NAD+/FAD) that carry the electrons to the electron transport chain, which produces a proton gradient across the membrane (PMF) that is necessary to synthesize ATP from ADP via ATP synthase (utilizing either oxygen or alternative terminal electron acceptors). The recycling of cofactors from electron transport for continued utilization in the TCA cycle is essential for continued carbon metabolism. (4) Malate is a product of the TCA cycle and an essential precursor for O1-antigen biosynthesis. One potential result of weakened PMF is a reduction in pools of carbon metabolites such as malate. (5) Reduction in the availability of O1-biosynthetic enzymes and putative reductions in malate pools result in a decreased capacity to synthesize O1-antigen. (6) In the conditions tested, O1-depletion occurs in the context of ToxT-mediated activation of a canonical *V. cholerae* pathogenesis transcriptional cascade.

To establish a thorough understanding of phage defense as it has evolved in native contexts, the interplay of many systems must be considered as a holistic picture. As the discovery and appreciation of novel phage defense systems rapidly accelerate, a more underappreciated route of inquiry dissecting the intertwined nature of phage defense and bacterial physiology has also recently gained appreciation. Reversible environmental adaptation conferring phage resistance was recently described in an *E. coli* mouse model, where differential expression of biofilm genes and O-antigen ligase in susceptible bacterial hosts limited phage predation in the intestine (59). *Streptomyces* species have been shown to convert to a cell-wall-deficient state when exposed to environmental pressures, including phage infection, and *E. coli* and *Bacillus subtilis* cultured in an osmoprotective environment also convert to a cell-wall-deficient state to resist phage infection by shedding the phage receptor (60). The work presented here demonstrates a related phenomenon in *V. cholerae*, where exposure to signals relevant to the intestinal environment transiently alters the availability of O1-antigen decoration on LPS, negatively impacting the adsorption of phages that require the O1-antigen as a receptor. We investigated the cause of this reduced O1-antigen production and identified several routes by which the decrease may occur, including a reduction in O1-biosynthetic enzyme production and global changes in gene expression, indicating changes in the availability of protein translation and central carbon metabolism components required for constructing O1-antigen subunits. We also identified the essentiality of the weak acid tolerance system *cadABC* in the production of O1-antigen in conditions mimicking the intestinal environment (Fig. 5).

This study is the first to describe altered transcription of O1-biosynthetic genes in response to intestine-associated stimuli coupled with the activation of the ToxR virulence transcriptional cascade. *In vivo* transcriptomic experiments in animal models did not detect differential expression of the O1-biosynthetic cluster (45). Previous characterizations of *V. cholerae’s* response to bile acids and anaerobicity individually were focused on relatively limited microarray (35) and 2D gel proteomic studies (61), respectively. While our work is the first (to our knowledge) to apply RNA sequencing to assess the synergistic effects of bile and anaerobicity on the *V. cholerae* transcriptome, our observations align well with previous analyses of these environmental factors and allowed for rich contextualization of some subtle trends in our transcriptomic data (see Text S1). Reminiscent of what we observed in anaerobic bile conditions, previous transcriptional analyses of *V. cholerae* in mouse and rabbit intestinal models identified the upregulation of pathogenesis, transport, and regulatory genes (62) coupled with transcriptional changes to fatty acid and glycerol metabolism, and activation of cryptic colonization factors in VPI-2 (*Vibrio* pathogenicity island-2), but no transcriptional changes in the O1-biosynthetic cluster (45). One potential explanation for this discrepancy could be differences in environmental structure. Growing bacteria in liquid culture represents a physically homogeneous mixture, while the intestinal environment is spatially complex. Spatial complexity has been shown to play a role in the stable coexistence of phages and bacteria (63) and is likely to contribute to the development of subpopulations with different transcriptional profiles. Such nuances would be lost in averaging transcriptome data for the entire population harvested from animal models. This could also help contextualize the surprising observation of O1-antigen reduction in anaerobic bile conditions considering the essentiality of the O1-antigen for intestinal colonization. It is possible that only some *V. cholerae* in an *in vivo* gut environment respond to collective bile, low pH, and anaerobic signals by reducing O1-antigen-decorated LPS, while other subpopulations experience different intensities of these signals and produce a different transcriptional response. This would allow for some subpopulations to express the optimal amount of O1-antigen for intestinal colonization, while other subpopulations potentially sacrifice some degree of colonization efficiency for protection from phage predation. Alternatively, O1-antigen expression may be important for the establishment of intestinal colonization but more dispensable later during infection, allowing for a population to efficiently colonize and then shift to a more phage-resistant state after initial establishment.

Previous work has also described transcriptional activation of *cadA* in rabbit and mouse cholera models (58). Although there was no observed colonization defect in a *cadA* mutant strain, there was an observed colonization advantage for acid-adapted *V. cholerae*, in which the *cadABC* system plays a pivotal role. We observed a very limited amount of O1-antigen produced by the *cadA* and *cadC* mutants in anaerobic bile conditions, which would be expected to significantly reduce colonization efficiency. Because previous observations of *cadA* mutants *in vivo* did not indicate colonization deficiency, this further supports the hypothesis that the *in vivo* environment provides niches with a variety of conditions that allow for *V. cholerae* to thrive with different degrees of O1-antigen production. We also observed a pH-dependent depletion of O1-decorated LPS under both organic acid stress and inorganic acid stress in the absence of weak acid tolerance, consistent with the previously identified role of *cadA* in the *V. cholerae* acid tolerance response to both types of acid (58). Overall, we identified that weak acid tolerance via *cadABC* is required for O1 biosynthesis under anaerobic bile conditions, suggesting that *cadABC* upregulation observed *in vivo* may be related to enhancing colonization efficiency by supporting O1-antigen biosynthesis. The requirement for *cadABC* could be due to the differential accumulation of metabolic byproducts in bile and the absence of oxygen that feed into O1 biosynthesis, or due to an undiscovered transcriptional connection between the regulation of weak acid tolerance and O1 biosynthesis.

While we identified several potential mechanisms by which O1-antigen production could be decreased in anaerobic bile conditions, we have yet to elucidate a direct link between O1-decoration and weak acid tolerance mediated by *cadABC*. In general, bacterial acid tolerance systems have been hypothesized to play essential roles in the maintenance of proton motive force (PMF) in extreme pH conditions (64). In *V. cholerae*, the hydrochloric-acid-specific tolerance system (*clcA*) relies on tightly regulated expression of chloride channels that exchange intracellular chlorine for hydrogen ions to avoid toxic membrane hyperpolarization (termed an “electrical shunt”). These channels must be quickly silenced in more alkaline pH conditions to avoid PMF disruption and may act in tandem with amino acid decarboxylation acid tolerance systems like *cadABC* (64, 65). The impact of anaerobic bile conditions on PMF is unclear, but there is a hypothetical role for *cadABC* activity in the maintenance of PMF and a logical link to O1 biosynthesis. CadA decarboxylates lysine and consumes protons in the process, and CadB consumes protons while importing lysine. These activities can increase cytoplasmic and periplasmic pH. If there is more decarboxylation of cellular lysine, the cytoplasmic pH neutralizes more rapidly and produces a stronger PMF. PMF is required for energy production via ATP synthase in the last step of cellular respiration. Energy production from ATP synthase also recycles metabolic cofactors (e.g., NAD+/FAD), which allows for continued production of central carbon metabolism components and ATP. In anaerobic conditions, *V. cholerae* likely relies on anaerobic respiration with alternative electron acceptors for energy metabolism (66, 67). A weakened PMF can slow down the entire carbon cycle due to reduced turnover of cofactors (68). Components of central carbon metabolism serve as the building blocks for O1-antigen subunit biosynthesis (perosamine is synthesized from D-fructose 6-phosphate, and tetronate is synthesized from malate (48)), so alterations in the PMF could negatively impact the availability of these components for O1-antigen biosynthesis. We hypothesize that the *V. cholerae cadA* and *cadC* mutants produce even less O1-antigen in anaerobic bile conditions because they are unable to strengthen the PMF with weak acid tolerance activity, resulting in a further hindrance of central carbon metabolism and reduction of O1-subunit biosynthesis components (Fig. 5). Taken together, this work suggests a cryptic role for acid tolerance as a determinant of phage infection by proxy of supporting biosynthesis of the O1-antigen phage receptor.

Membrane surface modification in *V. cholerae* appears to be a common strategy to afford resistance to many different biological threats. *V. cholerae* responds to bile stimulus with a ToxR-regulon-mediated exchange of outer membrane porin OmpT for OmpU, a different porin with an important role in bile resistance (12). *V. cholerae* lipid A is also modified via glycine addition, facilitating resistance to antimicrobial peptides during intestinal pathogenesis (46). Exchange of OmpT and unmodified lipid A for OmpU and modified lipid A first occurs at the transcriptional level, but clearance of existing OmpT and unmodified lipid A in the outer membrane is achieved by the production of outer membrane vesicles (OMVs), which are extrusions of accumulated phospholipids, LPS, and proteins from the outer membrane (69). This OMV-mediated surface exchange is also proposed to confer phage resistance: OMVs can act as decoys carrying phage receptors where phage may bind, interfering with receptor binding on viable *V. cholerae* cells. This effect has been demonstrated with vibriophages including ICP1, where exposure to high concentrations of OMVs modestly reduced phage titers (70). With the caveat that we did not observe differential expression of genes involved in OMV production in our RNA-seq analysis, OMV-mediated surface exchange coupled with decreased O1-antigen biosynthesis (which is a likely consequence of the observed depletion of O1-biosynthetic proteins (Fig. 3b through d) could result in the reduction of O1-decorated LPS we observed from *V. cholerae* cells in anaerobic bile culture. This would not only allow for the production of decoys but also provide a mechanism for shedding a substantial amount of phage receptor from the cell surface, hypothetically adding another mode of OMV-mediated phage resistance. However, it is currently unknown if OMV production plays a role in the reduction of O1-decorated LPS.

This work explores a physiological response to signals from *V. cholerae*’s natural environment that reduces O1-antigen decoration on LPS, an essential moiety for efficient intestinal colonization and for lytic phage predation. Studying the impact of these signals isolated from the *in vivo* environment allowed us to capture direct and relatively homogeneous responses to bile and anaerobicity that would not be possible in the heterogeneous *in vivo* intestinal environment. This response afforded protection against phages targeting the O1-antigen as a phage receptor, such as ICP1, without eliminating the antigen altogether. Reduction in O1-decorated LPS appeared to be a transient response to bile and anaerobicity, which are signals that are transiently encountered by *V. cholerae* while infecting a human host. As *V. cholerae* travels through the diverse “biomes” of the human digestive tract, the bacteria can temporarily alter their surface composition to respond to environmental changes without permanent disruption of genetic sequence. Such transient reduction in phage susceptibility could help explain why we observe both phages and phage-susceptible *V. cholerae* in cholera patient stool. The results underscore the importance of interrogating biological interactions in their environmental context. Incorporating environmental signals into standard laboratory culture conditions is not equivalent to studying complex environmental frameworks, but it allows for the impact of such signals to be interrogated in detail. The environmental context appears to be particularly important for predatory biological interactions like phage infections, which drive the rapid evolution of both phage and bacteria in nature.

## MATERIALS AND METHODS

### Strain construction

*V. cholerae* mutant strains with spectinomycin marker genes were constructed from E7946 (wild-type background) via natural transformation of linear PCR products (71) that were synthesized by splicing using overlap extension PCR (72) containing a spectinomycin resistance cassette or desired insertion sequence with 1 kB homologous sequence flanking each side. Natural transformants were selected on LB supplemented with spectinomycin (100 µg/mL) for knockouts or kanamycin (75 µg/mL) for insertions in the *lacZ* locus, and modifications were verified by Sanger sequencing. *V. cholerae* marker-less mutants were constructed by the pCVD442-lac method as previously described (24).

### Bacterial and phage growth conditions

*V. cholerae* E7946 and derivatives (a complete list of strains can be found in Table S1) were cultured from 20% (vol/vol) glycerol stocks stored at −80°C by streaking onto LB agar plates incubated at 37°C overnight. Plates were used to inoculate LB liquid media (Fisher Scientific), and liquid cultures were incubated at 37°C with aeration on a rotating shaker at 40 rpm. Bile-supplemented cultures contained 0.5% (wt/vol) purified dehydrated ox bile (Millipore-Sigma) in LB unless otherwise specified. Anaerobic media was degassed overnight and aliquoted into Hungate culture tubes (Chemglass Life Sciences CLS420801) in an anaerobic chamber (Coy Lab Products, 2.3% hydrogen, <30 ppm oxygen). Growth assays were conducted by growing cultures to saturation in aerobic LB, then back diluting to OD_600_ = 0.05 and allowing for growth in all media conditions for either 2 hours or overnight (~16 hours). Anaerobic media was inoculated with a small volume of saturated aerobic culture to an OD_600_ = 0.05 (20–50μL per 5 mL culture) diluted in 200 µL of LB and injected through the rubber stoppers with a 23 g syringe. For strains with chromosomal expression constructs, cultures were grown to saturation aerobically in LB supplemented with 1 mM isopropyl β-D-1-thiogalactopyranoside (IPTG) and 1.5 mM theophylline, then back diluted as described above in media containing the same concentrations of IPTG and theophylline. Cultures were supplemented with varying amounts of a 1M liquid stock of malic acid, L-lysine, L-arginine (Millipore-Sigma), hydrochloric acid, or sodium hydroxide (Fisher Scientific) to the designated concentration or final media pH.

Phage strains were propagated via soft agar overlay method on wild-type *V. cholerae* (for ICP1 and ICP3) and Δ*wbeL* (O1-) *V. cholerae* (for ICP2). Briefly, *V. cholerae* strains were grown to an OD_600_ = 0.3, infected with phage for 10 min, and then mixed in 3 mL molten 0.5% (wt/vol) agar in LB. LB agar was allowed to solidify at room temperature, then incubated at 37°C for 6 hours. Phages were harvested in STE buffer (1M NaCl, 200 mM Tris-HCl, 100 mM EDTA) overlayed on confluent lysis plaque plates, rocking overnight at 4°C on a platform shaker at 12 rpm. Buffer was then collected, chloroform-treated, and cleared by centrifugation at 5,000 × *g* for 10 min. Phages in the cleared lysate were then concentrated to a high titer by polyethylene glycol precipitation as described (73).

### LPS purification

Bacteria were grown as described in “Bacterial and phage growth conditions” section, and pellets were harvested via centrifugation at 5,000 × *g* for 3 min. LPS was purified via hot aqueous phenol extraction method (74): pellets were lysed in 200 µL lysis buffer [2% (vol/vol) 2-mercaptoethanol (BME), 10% (vol/vol) glycerol in 0.1M tris-HCl, pH 6.8], boiled for 15 min, then treated with 25 units (5 µL) RNase-A, 25 units (5 µL) DNase-I, and 10 units (10 µL) Proteinase K overnight at 37°C. Following overnight incubation, samples were incubated at 59°C for 4 hours. Next, an equal volume of cold tris-saturated phenol (pH 6.8) was added, and samples were mixed and incubated at 65°C for 15 min. Samples were then treated with 2.5 volumes cold diethyl ether, mixed thoroughly by inversion, then phase separated by centrifugation at 20,000 × *g* for 10 min. The organic phase was extracted, and the phenol extraction process was repeated a second time or until the organic phase was clear. Growth assays and purifications were performed in biological triplicate.

### LPS silver stain gels

Purified LPS was mixed with Lamelli sample buffer (Bio-Rad) to a 1× final concentration, boiled for 10 min, then run alongside a standard serial dilution of LPS purified from aerobically grown wild-type *V. cholerae* on 4–12% Bis-Tris Criterion XT gels (Bio-Rad) at 165 V for 40 min. Gels were washed with water and then fixed overnight with rocking at 4°C in a gel fix solution on a platform shaker at 12 rpm [40% (vol/vol) ethanol and 10% (vol/vol) acetic acid]. Gels were stained the following day using the sensitizing and staining solutions from the SilverQuest Silverstain kit (Invitrogen) followed by development with a modified developer [30% (wt/vol) sodium carbonate and 0.05% (vol/vol) formaldehyde] and stop solution [5% (vol/vol) acetic acid]. Gels were then imaged on an EZ Dock gel imager (Bio-Rad) with the white light sample tray. Image quantification was conducted in ImageJ. The intensity of a standard serial dilution of *V. cholerae* LPS (total lane intensity including both O1 and lipid A/core components) was used to generate a standard curve and calculate an intensity-standardized volume for each sample, then a second gel with those volumes of purified LPS were run and silver stained for final images and image analysis.

### Adsorption assays

*V. cholerae* cultures were grown as described in “Bacterial and phage growth conditions” section. OD_600_ was measured and used to calculate a sample volume equivalent to OD_600_ = 0.3 in 1 mL. Samples were pelleted by centrifugation at 5,000 × *g* for 3 min, spent media was aspirated, and the pellet was washed with 1 mL LB, re-pelleted by centrifugation, then resuspended in 1 mL LB supplemented with 10 mM magnesium chloride. Samples were heat killed at 55°C for 10 min (complete killing was confirmed by plating total volume post-treatment), cooled at room temperature for 5 min, then infected with phage at an MOI = 0.01 (MOI = multiplicity of infection, calculated based on the number of viable cells in the sample volume pelleted prior to heat killing). Infected samples were incubated at 37°C with aeration for 30 min on a rotating shaker at 40 rpm to allow for complete adsorption. Next, samples were treated with 20 µL of chloroform, vortexed, and then separated by centrifugation at 5,000 × *g* for 15 min. Lysate was then serially diluted and used in plaque assays with *V. cholerae* grown to an OD_600_ = 0.3. A parallel control was included with no cells (magnesium-supplemented LB heated in the heat-kill treatment prior to phage addition) to enumerate the exact phage input titer. Experiments were conducted in technical duplicate and biological triplicate.

### Growth curves

*V. cholerae* cultures were grown to OD_600_ ~1 in LB at 37°C with aeration on a rotating shaker at 40 rpm. Cultures were then back diluted to OD_600_ = 0.05 in aerobic or anaerobic media, with or without 0.5% (wt/vol) bile acid supplementation. Anaerobic cultures were quickly aliquoted into a clear 96-well plate and then sealed thoroughly with an optical seal (Bio-Rad). Aerobic cultures were similarly added to the other half of the plate, which remained unsealed. OD_600_ was measured over time in the 96-well plate shaking at 37°C in a plate reader (SpectraMax i3x). For paired CFU samples, separate cultures in aerobic and anaerobic tubes were started in parallel and harvested at the designated timepoints throughout the experiment. Growth curves were performed in technical and biological triplicate, a representative technical triplicate average is displayed in Fig. 1i, and biological replicates are displayed in Fig. S2H.

### Recovery assay

*V. cholerae* cultures were grown overnight in anaerobic bile conditions as described in “Bacterial and phage growth conditions” section. Cultures were harvested by centrifugation at 5,000 × *g* for 3 min, spent media was aspirated, and cell pellets were resuspended in an equal volume of fresh aerobic LB media. Cultures were then incubated at 37°C with aeration on a rotating shaker at 40 rpm, and samples were taken at the designated timepoints to harvest LPS or conduct adsorption assays. Recovery assays were performed in technical duplicate and biological triplicate.

### RNA sequencing and analysis

#### RNA isolation

Cultures were grown as described in “Bacterial and phage growth conditions” section, and cell pellets (approximately 8 × 10^7^ CFU) were harvested by centrifugation at 5,000 × *g* for 3 min. Pellets were resuspended in 200 µL TRI Reagent (Millipore-Sigma) and incubated for 5 min at room temperature before the addition of 40 µL chloroform. Samples were mixed thoroughly, incubated for 10 min at room temperature, then phase separated by centrifugation at 12,000 × *g* for 15 min at 4°C. The aqueous phase was extracted and mixed thoroughly with 110 µL isopropanol and 11 µL 3M sodium acetate (pH 7.4) and then incubated at room temperature for 10 min. Samples were phase separated again by centrifugation as described above, and then, the aqueous phase was extracted, washed with 1 mL 75% (vol/vol) ethanol, and centrifuged again to pellet RNA. Ethanol was aspirated, and the residual was evaporated at 65°C prior to pellet resuspension in 40 µL DEPC treated water (Growcells.com). Samples were DNase treated with DNase-I (RNase free) (Invitrogen) at 37°C for 20 min.

#### RNA sequencing

Approximately 1 µg of RNA [A_260_/A_230_ 1.8–2.2 on NanoDrop (Thermo), RIN >6] per sample was submitted to the Microbial Genome Sequencing Center for 2 × 150 paired-end sequencing, and RNA sequencing was carried out according to the Microbial Genome Sequencing Center protocols. rRNA was depleted from the samples using the Ribo-Zero Plus kit (Illumina) following the manufacturer’s protocol. Library preparation was performed using Illumina’s Stranded Total RNA Prep Ligation with 10 bp unique dial indices (UDI). Sequencing was done on a NextSeq 2,000 instrument, giving 2 × 51 bp reads. Demultiplexing, quality control, and adapter trimming were performed with bcl-convert (v4.0.3).

#### RNA sequence analysis

Demultiplexed and trimmed fastq files were mapped to the *V. cholerae* E7946 reference genomes (Accession Nos.: NZ_CP024162 and NZ_CP024163) using bowtie2 (v 2.3.5.1), and the resulting .bam files were sorted using samtools (v 1.9). Sorted .bam files were assembled into transcripts with Stringtie (v 2.1.7) with options (-eB) to output in ballgown format. Ballgown files for all samples were converted into hypothetical read counts for each transcript using the Stringtie-associated prepDE.py python script. The resulting transcript count matrix was imported into DESeq2 (v 1.34.0) for further analysis in R.

To perform QC analysis and to determine what appropriate comparisons could be generated from the data set, the transcript count matrix was transformed according to the regularized log transformation (rlog) in DESeq2, and principal component analysis (PCA) was performed using PCAtools (v2.6). Dendrograms with heatmaps of the rlog transformed transcript count matrix were constructed using the R package pheatmap (v 1.0.12).

Differential expression analysis was performed with DESeq2, using the contrast function to generate pairwise comparisons of samples within a single timepoint according to their media conditions and aerobicity, to allow for a single-factor DESeq design formula. Genes were considered to be differentially regulated with a log_2_ fold change of ±1.5 and above a minimum adjusted *P*-value of 0.05. Volcano plots were generated from DESeq values in Microsoft Excel (Data Sheet 1), and differentially expressed genes were colored according to prediction function from a combination of Gene Ontology prediction (75, 76) and manual curation.

### Western blot

*V. cholerae* cultures were grown overnight aerobically and anaerobically in LB with and without 0.5% (wt/vol) bile acid supplementation. OD_600_-standardized pellets were harvested by centrifugation at 5,000 × *g* for 3 min, washed once with cold PBS, then resuspended on ice-cold lysis buffer [50 mM Tris, 150 mM NaCl, 1 mM EDTA, 0.5% (vol/vol) Triton X-100, 1× Pierce Protease Inhibitor Mini Tablet (Thermo)]. Protein concentration was determined using a Pierce BCA Protein Assay Kit (Thermo), and 30 µg total protein per sample was mixed with Laemmli sample buffer (10% (vol/vol) 2-mercaptoethanol added to a final concentration of 1×). Samples were boiled at 99°C for 10 min, run on Any-kD TGX-SDS-PAGE gels (Bio-Rad) at 200 V for 25 min, and then transferred onto nitrocellulose membranes using a Transblot Turbo Transfer system (Bio-Rad). Following overnight blocking in 5% (wt/vol) dehydrated milk rocking on a lateral shaker at 12 rpm, membranes were incubated with a primary rabbit anti-FLAG antibody (Invitrogen) diluted 1:1,500 for 3 hours. Signal detection was conducted with a goat anti-rabbit-HRP secondary antibody followed by development with Clarity ECL Substrate (Bio-Rad). Blots were imaged on Chemidoc XRS Imaging System (Bio-Rad). Band quantification was conducted using ImageJ. Western blots were conducted in biological triplicate.

### Phage infection survival assay

*V. cholerae* were grown overnight in triplicate in anaerobic bile culture as described in “Bacterial and phage growth conditions” section. Prior to infection, 200 µL of each culture was extracted with a syringe, serially diluted, and plated to quantify colony forming units (CFUs). A volume of ICP1 phage equivalent to a multiplicity of infection (MOI) of 2 (2 phage per bacterial cell, bacterial cell number estimated by optical density) was diluted in 200 µL LB and injected into the anaerobic bile overnight culture. Anaerobic tubes were returned to a 37°C incubator with rotation at 40 rpm. After 20 min, a 200 µL sample of infected culture was collected, serially diluted, and plated to quantify CFUs. Colonies at all countable dilutions were counted and averaged, and all three replicates per condition on a given day were averaged for a single biological replicate.

### Statistical analysis

All experiments were performed in technical duplicate and biological triplicate unless otherwise indicated. Statistical analysis was performed with Prism. Initial ANOVA (Brown-Forsythe and Welch) analysis was conducted to determine if there were statistically significant differences between groups. If a significant difference was found, a follow-up analysis was conducted using Dunnett’s T3 multiple comparisons analysis using the baseline condition (aerobic LB or standard pH) as the control group. If Dunnett’s T3 test returned a Type II error, alternative follow-up analysis was conducted with individual Welch’s *t*-test comparing each non-standard condition with the aerobic LB control.

## Data Availability

Transcriptomic data from the RNA sequencing experiment are deposited in the Sequence Read Archive under the BioProject accession PRJNA954450.

## SUPPLEMENTAL MATERIAL

Text S1 mbio.01985-23-s0001.docx Analysis of RNA-seq data.

Supplemental figures and table mbio.01985-23-s0002.pdf Figures S1–S12 and Table S1.

Supplemental data mbio.01985-23-s0003.xlsx Data Sheets S1–S3.

ASM does not own the copyrights to Supplemental Material that may be linked to, or accessed through, an article. The authors have granted ASM a non-exclusive, world-wide license to publish the Supplemental Material files. Please contact the corresponding author directly for reuse.
