# Supplementary material for: Adaptation to bile and anaerobicity limits Vibrio cholerae phage adsorption
Source: mBio. 2023 Oct 26;14(6):e01985-23. doi: 10.1128/mbio.01985-23 (PMC10746206; doi:10.1128/mbio.01985-23)
Supplement: Supplemental figures and table — Figures S1–S12 and Table S1. [file mbio.01985-23-s0002.pdf]

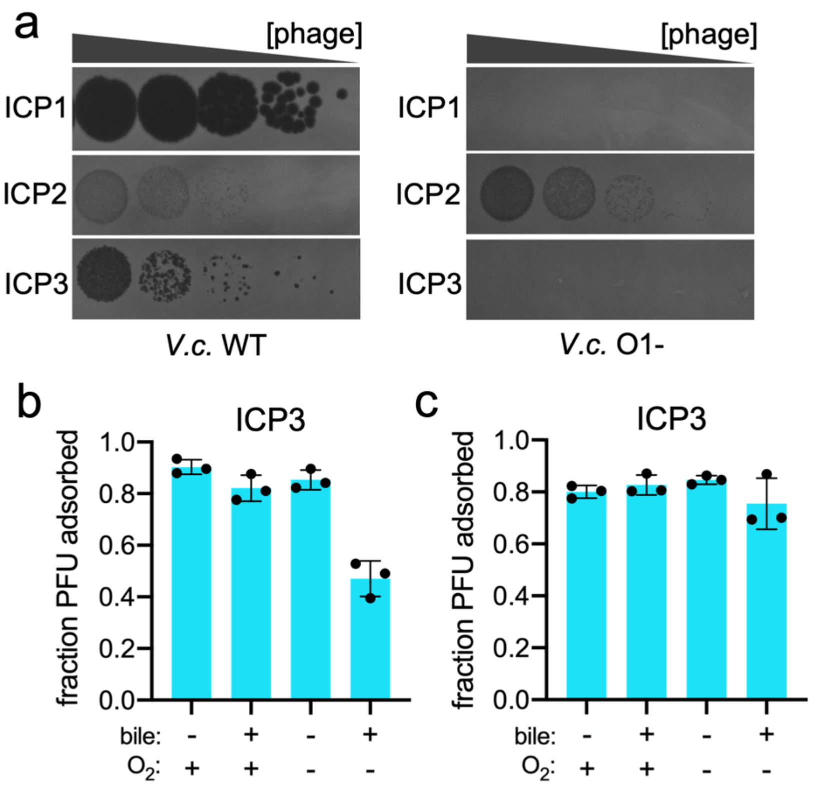

Supplementary Figure S1
(**a**) ICP1, ICP2, and ICP3 phage spot assays. Dark spots represent phage lysis and clearance of the *V. cholerae* host lawn (grey), for both wild-type (*V.c.* WT) and *V. cholerae* lacking the O1-antigen (*V.c.* O1-). Spots within a row represent 10-fold serial dilutions of the denoted phage. (**b**) Fraction of O1-dependent phage ICP3 adsorbed by *V. cholerae* cultured overnight in all combinations of culture conditions. (**c**) Fraction of ICP3 adsorbed by *V. cholerae* cultured for two hours in all combinations of culture conditions.

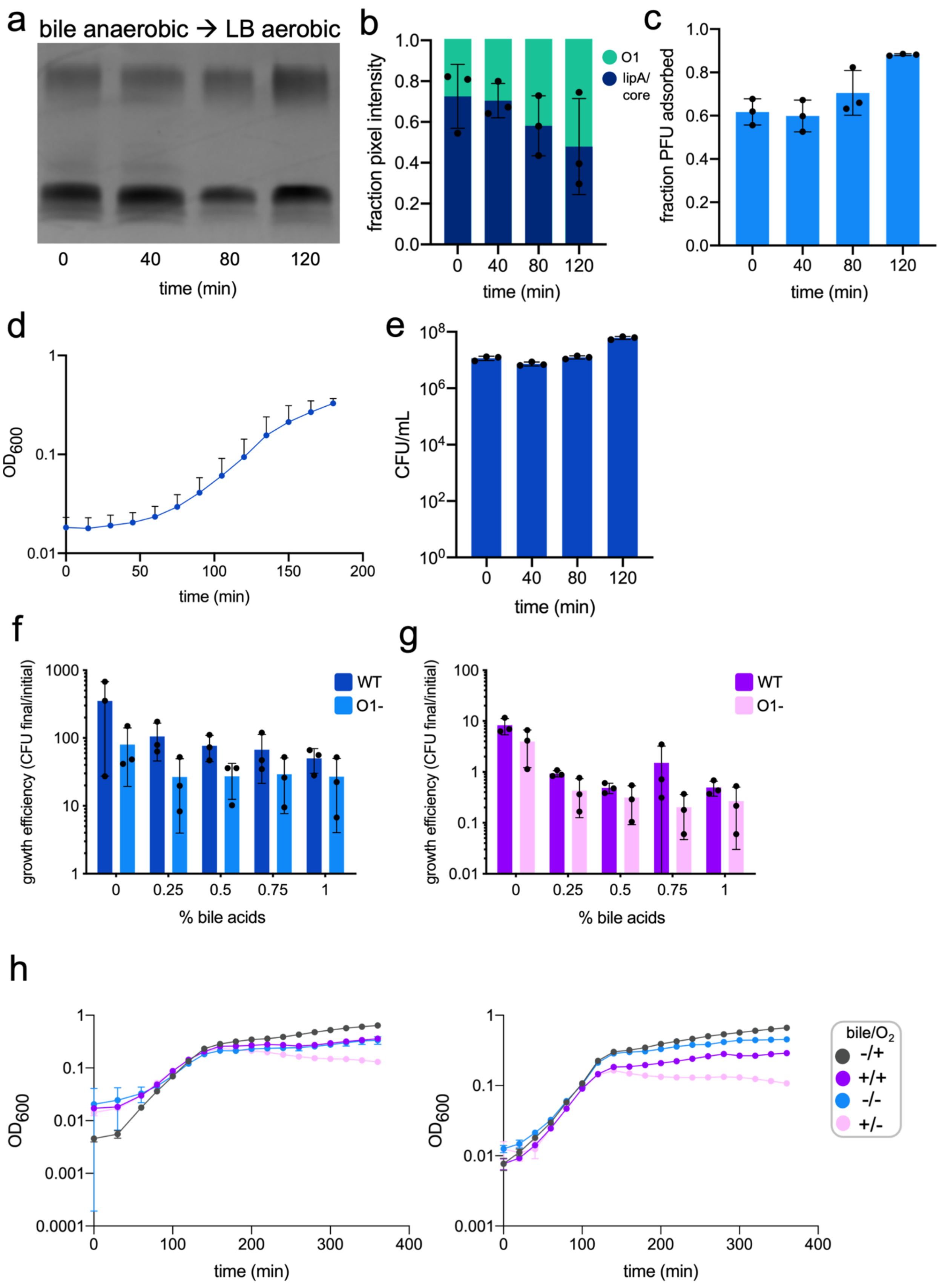

a
bile anaerobic → LB aerobic
0
40
80
120
time (min)
b
fraction pixel intensity
O1
lipA/core
time (min)
c
fraction PFU adsorbed
time (min)
d
OD600
time (min)
e
CFU/mL
time (min)
f
growth efficiency (CFU final/initial)
WT
O1-
% bile acids
g
growth efficiency (CFU final/initial)
WT
O1-
% bile acids
h
OD600
time (min)
bile/O2
-/+
+/+
-/-
+/-

Supplementary Figure S2

(**a**) Purified LPS silver stain of *V. cholerae* grown overnight in anaerobic bile conditions, then recovered in aerobic LB, time in minutes post-recovery. (**b**) Average fraction of pixel intensity contributed by O1 (light green) and lipid A/core (dark blue) quantifying LPS purification and silver stain replicates represented in (a). (**c**) Fraction of ICP1 adsorbed to *V. cholerae* anaerobic bile overnight cultures at timepoints during recovery in aerobic LB. (**d**) OD_{600} and (**e**) colony-forming units (CFU) of *V. cholerae* anaerobic bile overnight cultures recovering in aerobic LB. (**f**) Growth efficiency of wild-type (WT) and O1-antigen-null (O1-, Δ*wbeL*) *V. cholerae* grown aerobically in increasing concentrations of bile acids. Growth efficiency is calculated as $CFU_{final}/CFU_{initial}$. (**g**) same as (f) but in anaerobic conditions. (**h**) Biological replicates (each in technical triplicate) of growth monitored by OD_{600} over time in all combinations of culture conditions represented in Figure 1I.

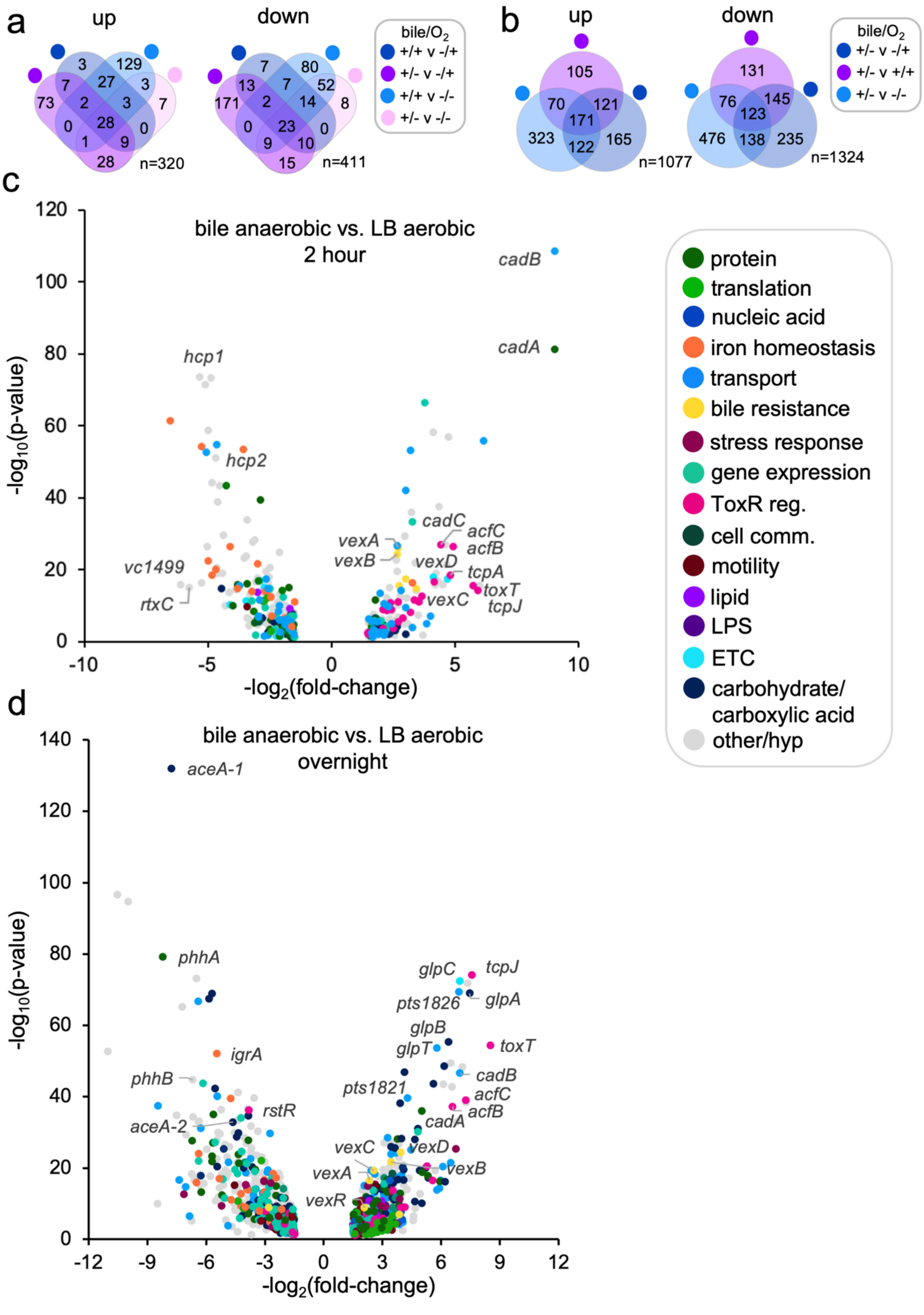
a
up
down
n=320
n=411
bile/O2
+/+ v -/+
+/- v -/+
+/+ v -/-
+/- v -/-
b
up
down
n=1077
n=1324
bile/O2
+/- v -/+
+/- v +/+
+/- v -/-
c
bile anaerobic vs. LB aerobic
2 hour
-log10(p-value)
-log2(fold-change)
cadB
cadA
hcp1
hcp2
vc1499
rtxC
vexA
vexB
cadC
acfC
acfB
vexD
tcpA
toxT
tcpJ
vexC
protein
translation
nucleic acid
iron homeostasis
transport
bile resistance
stress response
gene expression
ToxR reg.
cell comm.
motility
lipid
LPS
ETC
carbohydrate/
carboxylic acid
other/hyp
d
bile anaerobic vs. LB aerobic
overnight
-log10(p-value)
-log2(fold-change)
aceA-1
phhA
igrA
phhB
rstR
aceA-2
glpC
tcpJ
glpA
pts1826
glpB
glpT
toxT
cadB
acfC
acfB
pts1821
cadA
vexC
vexD
vexA
vexB
vexR

Supplementary Figure S3

(**a**) Venn diagram depicting differentially expressed genes shared between pairwise comparisons at two hours. "n" denotes total number of genes. (**b**) same as (a) at the overnight timepoint. (**c**) Two-hour and (**d**) overnight volcano plots of differentially expressed genes in anaerobic bile compared to aerobic LB conditions. Plots represent the same data as in Figure 2B-2C but with full gene ontology metabolic process category designation or curated category designation. Adjusted p-value $p \leq 0.05$ and fold-change $\geq$ +/-1.5 was considered significant. General categories in legend refer to gene ontology metabolic processes. 'ToxR reg.': ToxR regulon, 'cell comm.': cell communication, 'hyp': hypothetical.

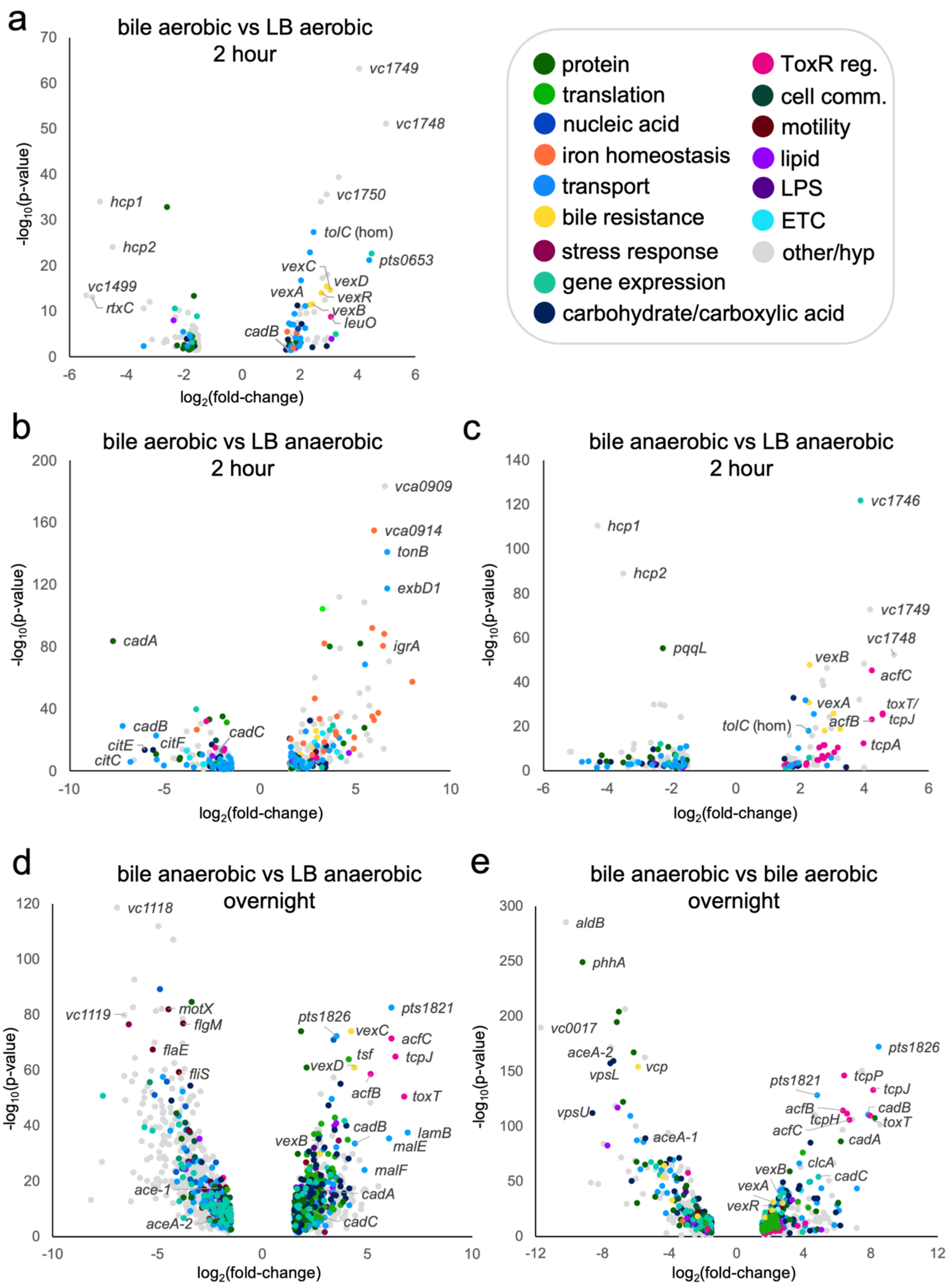
a
bile aerobic vs LB aerobic
2 hour
vc1749
vc1748
vc1750
hcp1
hcp2
tolC (hom)
pts0653
vexC
vexD
vexA
vexR
vexB
leuO
vc1499
rtxC
cadB
-log10(p-value)
log2(fold-change)
protein
translation
nucleic acid
iron homeostasis
transport
bile resistance
stress response
gene expression
carbohydrate/carboxylic acid
ToxR reg.
cell comm.
motility
lipid
LPS
ETC
other/hyp
b
bile aerobic vs LB anaerobic
2 hour
vca0909
vca0914
tonB
exbD1
igrA
cadA
cadB
cadC
citE
citF
citC
c
bile anaerobic vs LB anaerobic
2 hour
vc1746
hcp1
hcp2
vc1749
vc1748
pqqL
vexB
acfC
vexA
toxT/
tcpJ
acfB
tolC (hom)
tcpA
d
bile anaerobic vs LB anaerobic
overnight
vc1118
vc1119
motX
flgM
flaE
fliS
pts1821
pts1826
vexC
acfC
tsf
tcpJ
vexD
acfB
toxT
cadB
lamB
vexB
malE
malF
ace-1
cadA
aceA-2
cadC
e
bile anaerobic vs bile aerobic
overnight
aldB
phhA
vc0017
aceA-2
vcp
vpsL
vpsU
aceA-1
pts1826
pts1821
tcpP
tcpJ
acfB
cadB
tcpH
toxT
acfC
cadA
clcA
vexB
cadC
vexA
vexR

Supplementary Figure S4

(**a**) Volcano plot comparing differentially expressed genes in aerobic bile vs. aerobic LB at two hours. (**b**) Volcano plot comparing differentially expressed genes in aerobic bile vs. anaerobic LB at two hours. (**c**) Volcano plot comparing differentially expressed genes in anaerobic bile vs. anaerobic LB at two hours. (**d**) Volcano plot comparing differentially expressed genes in anaerobic bile vs. anaerobic LB overnight cultures. (**e**) Volcano plot comparing differentially expressed genes in anaerobic bile vs. aerobic bile overnight cultures.

For all plots, adjusted p-value $p \leq 0.05$ and fold-change $\geq$ +/-1.5 was considered significant. General categories in legend refer to gene ontology metabolic processes. 'ToxR reg.': ToxR regulon, 'cell comm.': cell communication, 'hyp': hypothetical.

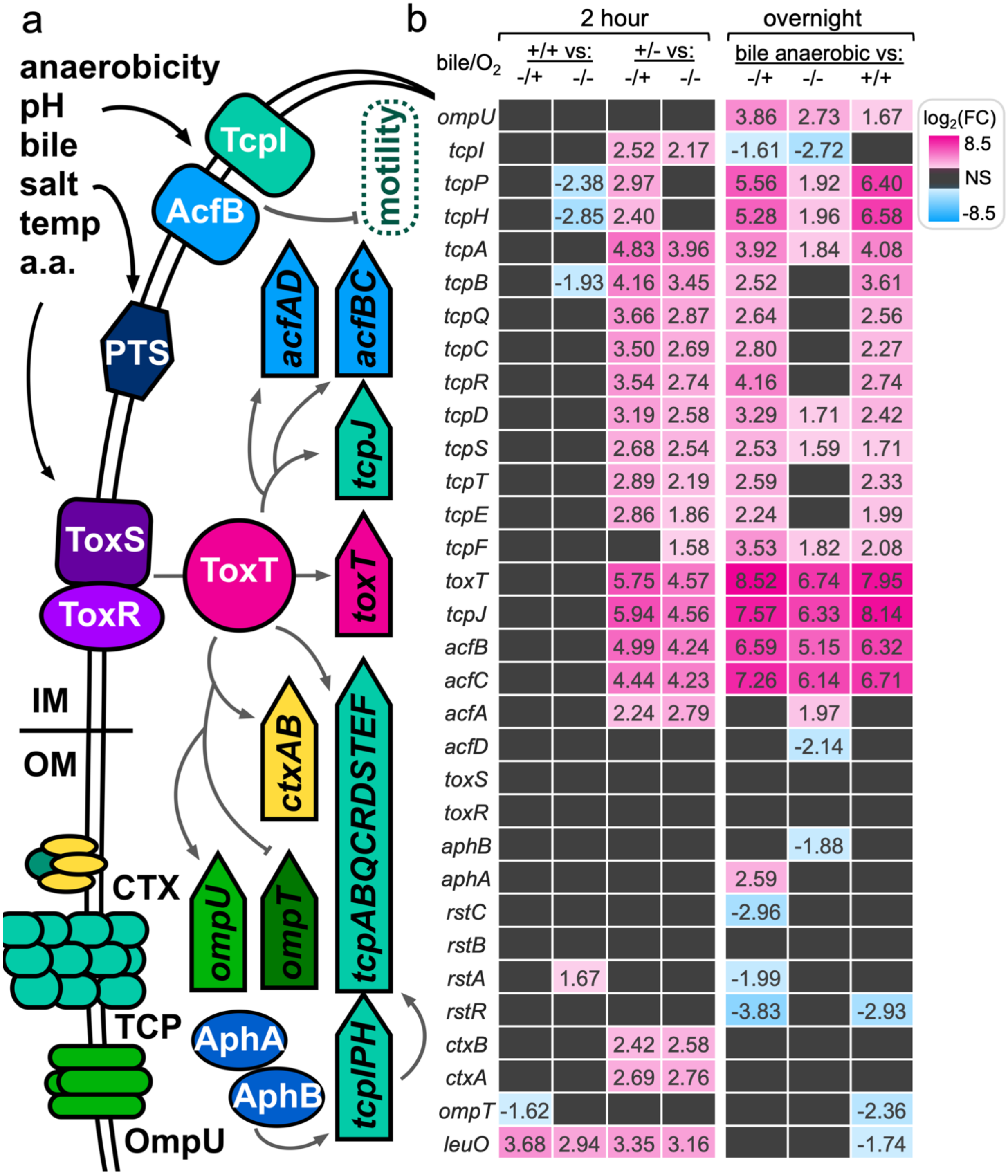

Supplementary Figure S5

(**a**) Schematic of select components of the *V. cholerae* toxR virulence regulon. Signals including anaerobicity, pH, bile, osmolarity, temperature ("temp."), and amino acids ("a.a.") are sensed by the ToxRS complex in the inner membrane ("IM"). Stimulation of ToxRS activates expression of *toxT*, which encodes the ToxT transcriptional activator. ToxT activates expression of *ctx* genes encoding the components of cholera toxin and *tcp* genes responsible for building the toxin-

(*Supp. Figure 4 cont.*) coregulated pilus (TCP) at the outer membrane ("OM"), as well as *acf* accessory colonization factor genes involved in a variety of pathogenesis related functions including repression of motility. The ToxR regulon also alters the composition of outer membrane porins by upregulating expression of *ompU* and downregulating *ompT*. Anaerobicity activates AphAB via a thiol-based switch mechanism. AphB is a transcriptional regulator that independently activates *tcpPH* expression. The PTS system senses environmental glucose availability and alters expression of transporters for alternative carbon sources. (refs. 12, 13) (**b**) Heatmap of log_2(fold-change) for genes in the ToxR regulon for two-hour and overnight culture comparisons. log_2(fold-change) values are listed in individual cells. Legend gives colorimetric approximation of fold-change, NS (black) denotes nonsignificant.

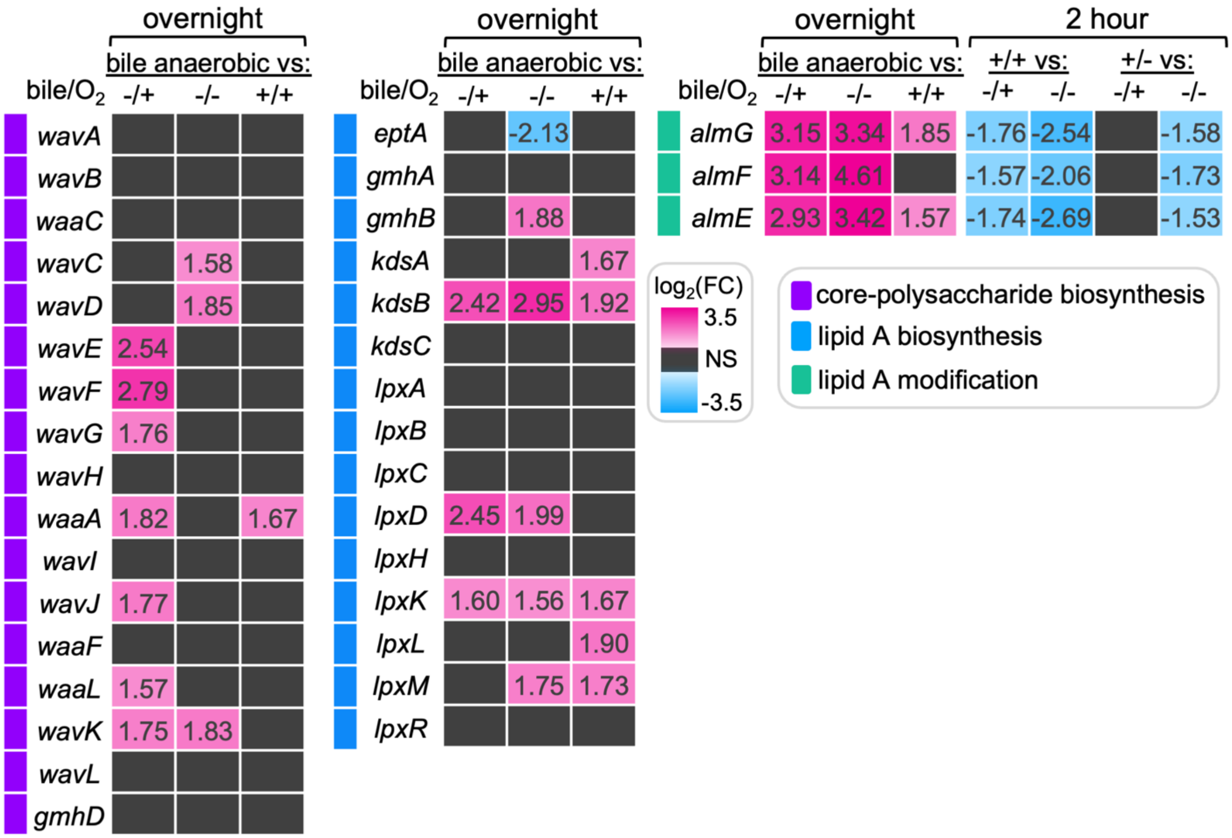

Supplementary Figure S6

Heatmap of log_2(fold-change) for genes involved in other LPS biosynthetic processes besides O1-antigen biosynthesis (categories on left) for overnight culture comparisons, and additionally at the two-hour timepoint for lipid A modification genes. log_2(fold-change) values are listed in individual cells and legend gives colorimetric approximation of fold-change, NS (black) denotes nonsignificant. Second legend indicates gene pathway. Two-hour lipid A/core-polysaccharide biosynthesis gene comparisons and LPS export genes (*lpt* system) were excluded because they were not differentially expressed.

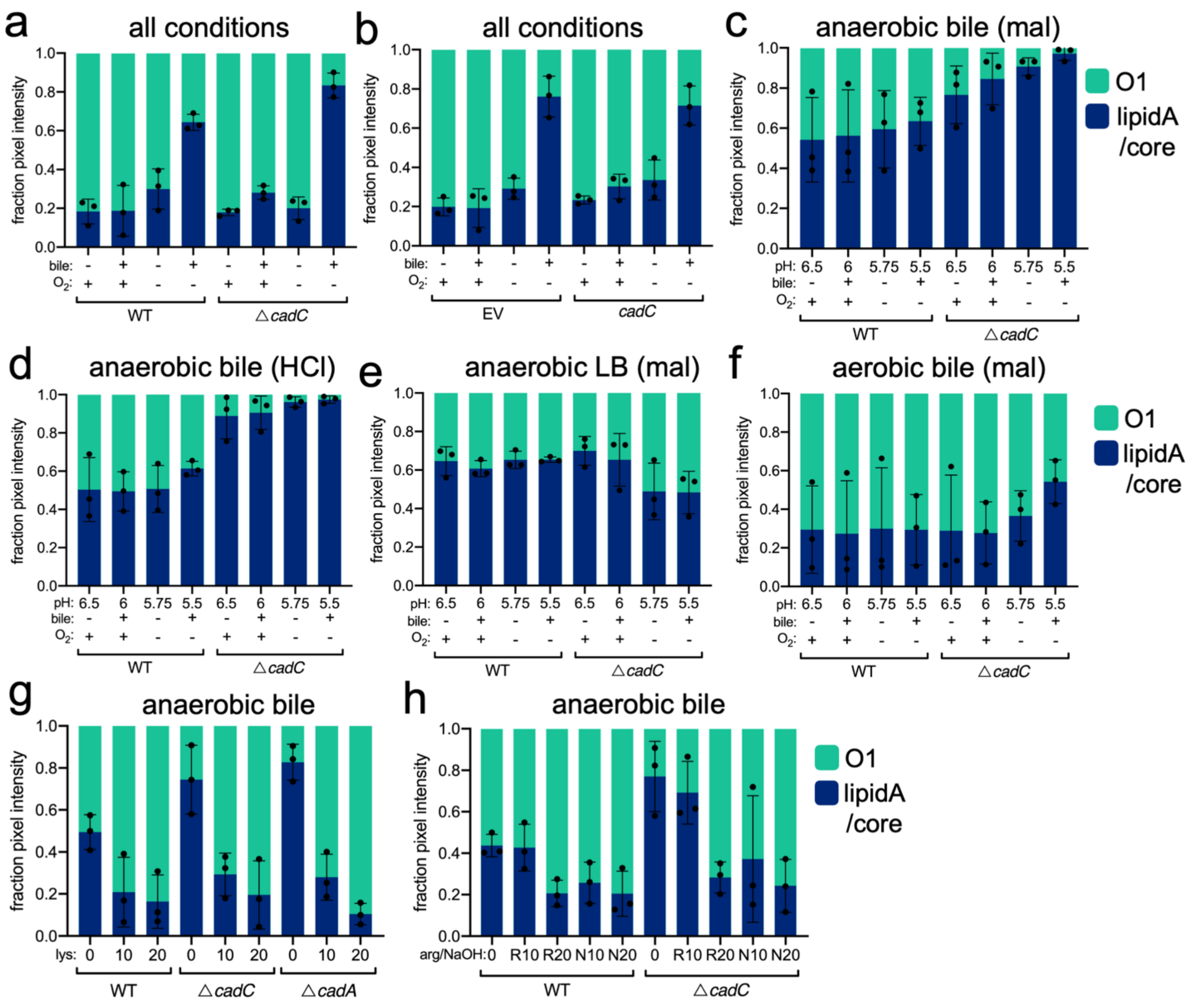

Supplementary Figure S7

Pixel intensity quantifications for replicate purified LPS silver stain gels (O1 = light green, lipid A/core = dark blue) for the following experiments: (**a**) wild-type (WT) and Δ*cadC V. cholerae* grown overnight in all combinations of culture conditions. (**b**) *V. cholerae* expressing an empty vector (EV) or *cadC* from the *lacZ* locus grown overnight in all combinations of culture conditions. (**c**) wild-type (WT) and Δ*cadC V. cholerae* grown overnight in anaerobic bile culture at low pH (reduced with malic acid). (**d**) wild-type (WT) and Δ*cadC V. cholerae* grown overnight in anaerobic bile culture at low pH (reduced with hydrochloric acid). (**e**) wild-type (WT) and Δ*cadC V. cholerae* grown overnight in anaerobic LB culture at low pH (reduced with malic acid). (**f**) wild-type (WT) and Δ*cadC V. cholerae* grown overnight in aerobic bile culture at low pH (reduced with malic acid). (**g**) wild-type (WT), Δ*cadC*, and Δ*cadA V. cholerae* grown overnight in anaerobic bile culture supplemented with L-lysine (lys, mM). (**h**) wild-type (WT), Δ*cadC*, and Δ*cadA V. cholerae* grown overnight in anaerobic bile culture supplemented with L-arginine (arg/R, mM) or pH adjusted with sodium hydroxide (NaOH/N).

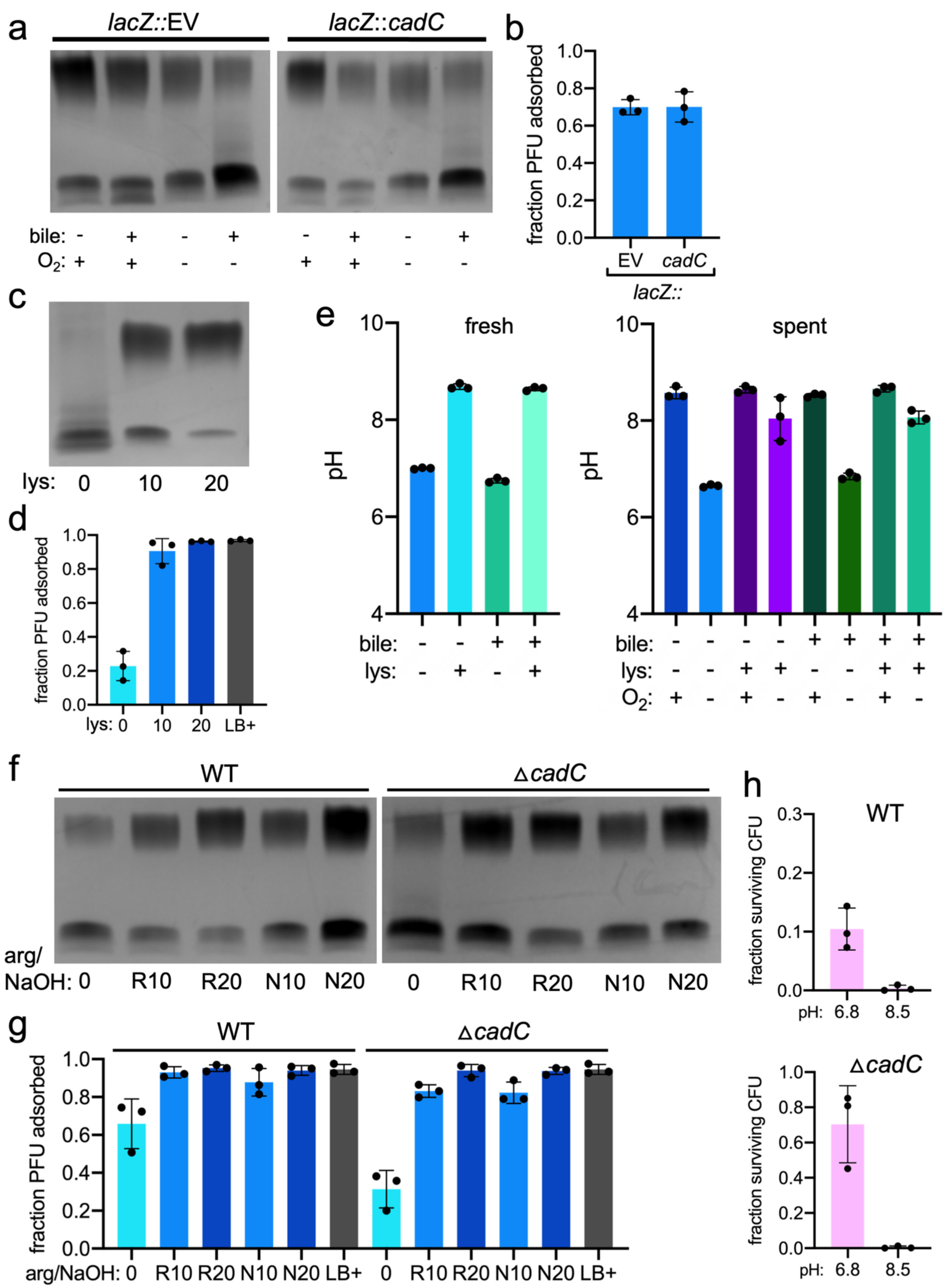
a
lacZ::EV
lacZ::cadC
bile: - + - + - + - +
O2: + + - - + + - -
b
fraction PFU adsorbed
EV
cadC
lacZ::
c
lys: 0 10 20
d
fraction PFU adsorbed
lys: 0 10 20 LB+
e
fresh
spent
pH
bile:
lys:
O2:
f
WT
ΔcadC
arg/
NaOH: 0 R10 R20 N10 N20 0 R10 R20 N10 N20
g
WT
ΔcadC
fraction PFU adsorbed
arg/NaOH: 0 R10 R20 N10 N20 LB+ 0 R10 R20 N10 N20 LB+
h
WT
ΔcadC
fraction surviving CFU
pH: 6.8 8.5

Supplementary Figure S8

(**a**) Purified LPS silver stain of *V. cholerae* grown overnight in all combinations of culture conditions synthetically induced to express *cadC* or an empty vector (EV) in the *lacZ* locus. (**b**) Fraction of ICP1 adsorbed to *V. cholerae* grown overnight in anaerobic bile culture, synthetically induced to express *cadC* or an empty vector (EV) in the *lacZ* locus. (**c**) Purified LPS silver stain of *V. cholerae* Δ*cadA* grown overnight in anaerobic bile culture supplemented with L-lysine (lys, mM). (**d**) Fraction of ICP1 adsorbed to *V. cholerae* Δ*cadA* grown overnight in anaerobic bile culture supplemented with lysine (lys, mM) or aerobic LB (LB+). (**e**) pH measurements of fresh (left) and aerobically/anaerobically spent (right, O_2 +/-) LB media with and without 0.5% bile acid supplementation and 20mM L-lysine supplementation. (**f**) Purified LPS silver stain of *V. cholerae* grown overnight in anaerobic bile culture conditions supplemented with L-arginine (arg/R, mM) or pH adjusted with sodium hydroxide to lysine/arginine equivalent (NaOH/N). (**g**) Fraction of ICP1 adsorbed to *V. cholerae* grown overnight in anaerobic bile culture conditions supplemented with L-arginine (arg/R, mM) or pH adjusted with sodium hydroxide to lysine/arginine equivalent (NaOH/N). LB+ denotes aerobic LB control. (**h**) Fraction of surviving wild-type (WT, top) and Δ*cadC* (bottom) *V. cholerae* colony forming units (CFUs) grown overnight in anaerobic bile culture at the indicated pH (adjusted with sodium hydroxide) following a single round of ICP1 infection at a multiplicity of infection (MOI) of 2.

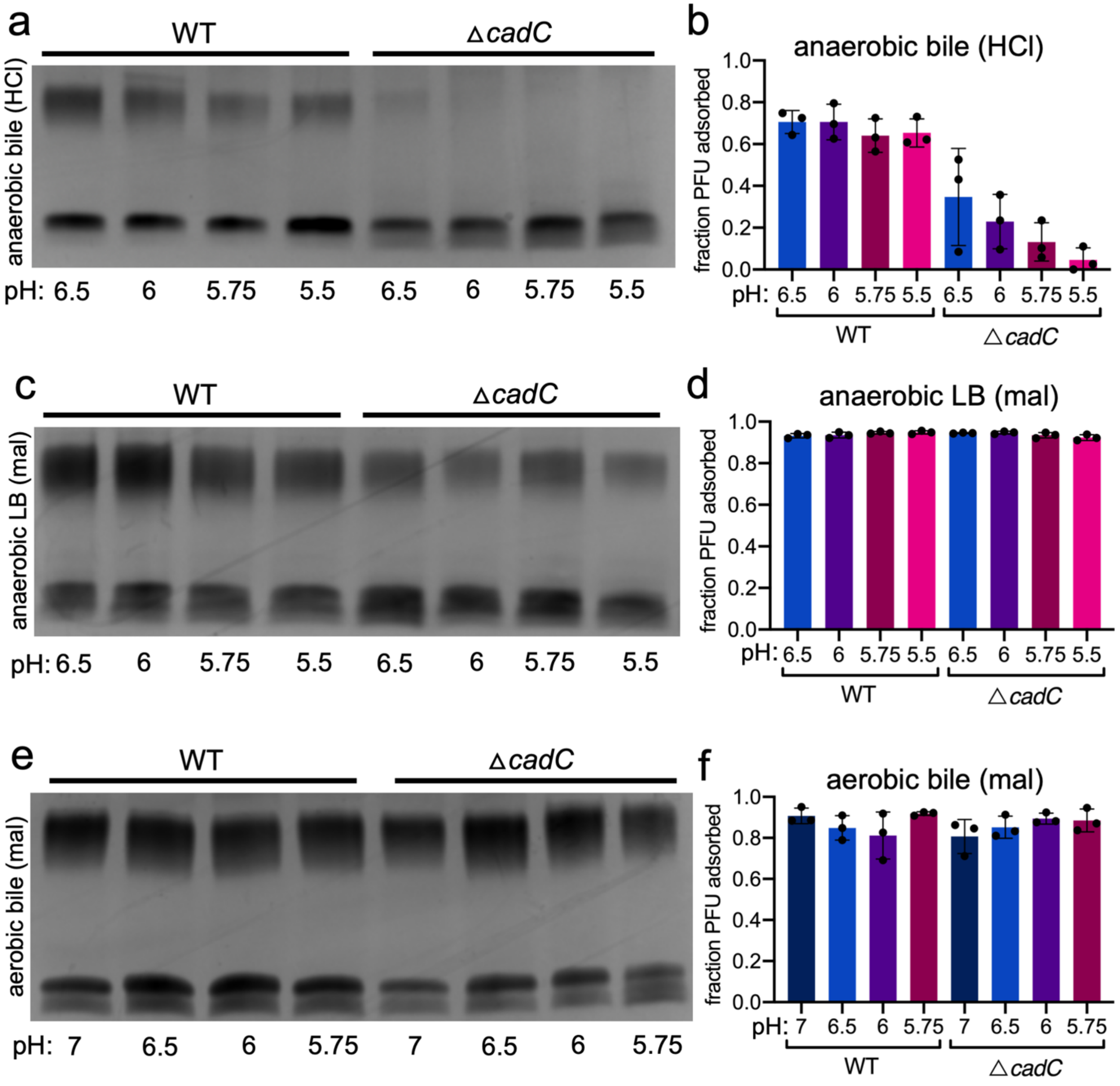

Supplementary Figure S9

(**a**) Purified LPS silver stain of *V. cholerae* grown overnight in anaerobic bile culture conditions, pH adjusted with hydrochloric acid. (**b**) Fraction of ICP1 adsorbed to *V. cholerae* grown overnight in anaerobic bile culture conditions, pH reduced with hydrochloric acid (HCl). (**c**) Purified LPS silver stain of *V. cholerae* grown overnight in anaerobic culture conditions, pH reduced with malic acid (mal). (**d**) Fraction of ICP1 adsorbed to *V. cholerae* grown overnight in anaerobic culture conditions, pH reduced with malic acid (mal). (**e**) Purified LPS silver stain of *V. cholerae* grown overnight in aerobic bile culture conditions, pH reduced with malic acid

(mal). (**f**) Fraction of ICP1 adsorbed to *V. cholerae* grown overnight in aerobic bile culture conditions, pH reduced with malic acid (mal).

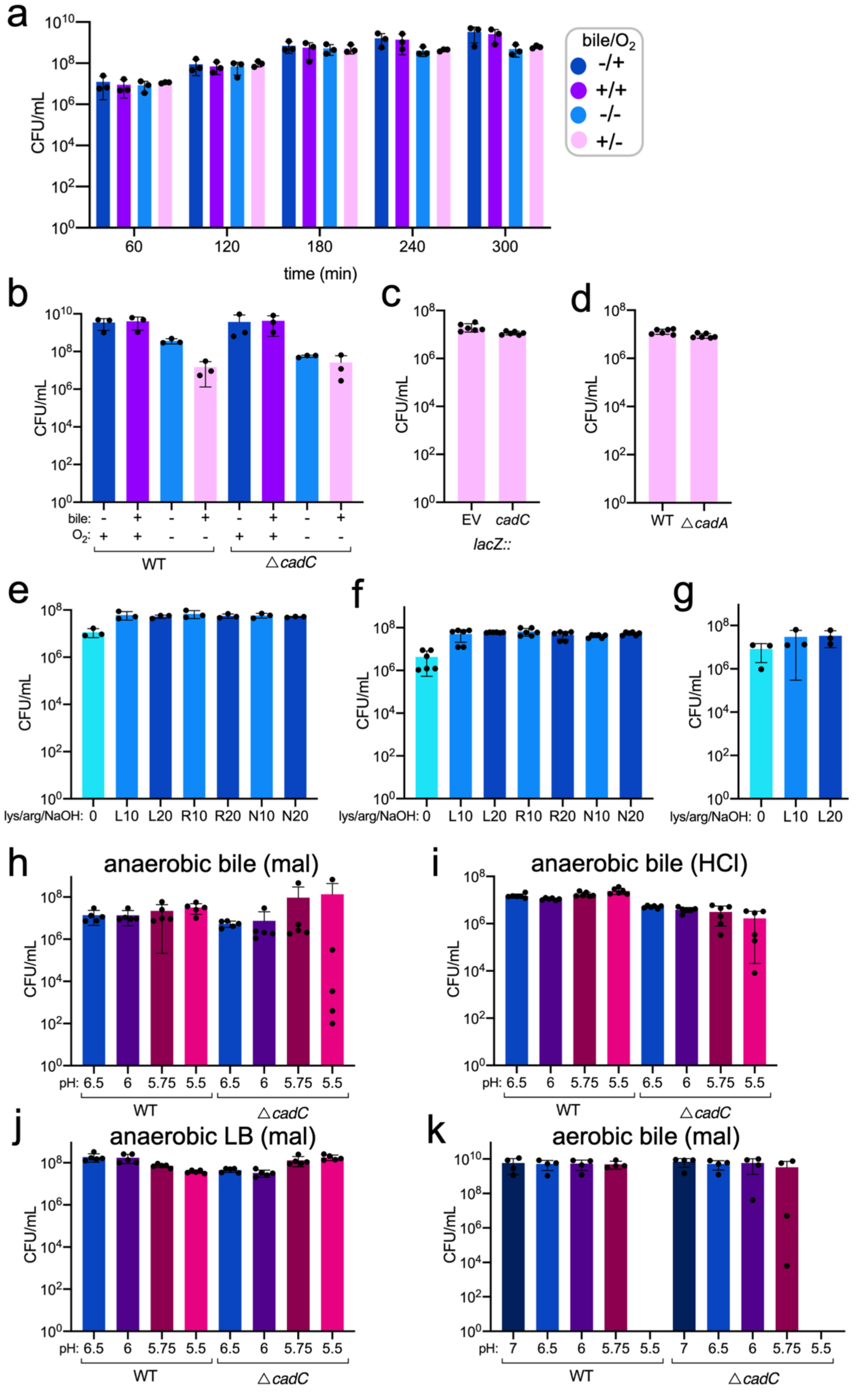

a
bile/O2
-/+
+/+
-/-
+/-
CFU/mL
time (min)
60
120
180
240
300
b
bile:
O2:
WT
△cadC
c
EV
cadC
lacZ::
d
WT
△cadA
e
f
g
lys/arg/NaOH: 0 L10 L20 R10 R20 N10 N20
h
anaerobic bile (mal)
i
anaerobic bile (HCl)
j
anaerobic LB (mal)
k
aerobic bile (mal)
pH: 6.5 6 5.75 5.5
pH: 7 6.5 6 5.75 5.5

Supplementary Figure S10

Colony forming units (CFU) quantified for the following experiments:
(a) wild-type *V. cholerae* grown aerobically or anaerobically with or without bile acid supplementation at the indicated time post-inoculation (in minutes). (b) wild-type (WT) and Δ*cadC V. cholerae* grown overnight aerobically and anaerobically (O_2+/-) in LB or LB supplemented with 0.5% bile (bile+/-). (c) *V. cholerae* grown overnight in anaerobic bile culture induced to express an empty vector (EV) or *cadC* from the chromosomal *lacZ* locus. (d) wild type (WT) and Δ*cadA V. cholerae* grown overnight in anaerobic bile culture. (e) wild-type *V. cholerae* grown overnight in anaerobic bile culture with lysine (lys/L), arginine (arg/R), or sodium hydroxide (NaOH/N) supplementation. The number next to the letter denotes mM concentration for lysine and arginine, pH matched to mM amino acid concentrations for sodium hydroxide. (f) *V. cholerae* Δ*cadC* grown overnight in anaerobic bile culture with lysine (lys/L), arginine (arg/R), or sodium hydroxide (NaOH/N) supplementation. The number next to the letter denotes mM concentration for lysine and arginine, pH matched to mM amino acid concentrations for sodium hydroxide. (g) *V. cholerae* Δ*cadA* grown overnight in anaerobic bile culture with lysine (lys/L). The number next to the letter denotes mM concentration. (h) Wild-type (WT) and and Δ*cadC V. cholerae* grown overnight in anaerobic bile culture, pH reduced with malic acid. (i) wild-type (WT) and and Δ*cadC V. cholerae* grown overnight in anaerobic bile culture, pH reduced with hydrochloric acid. (j) wild-type (WT) and and Δ*cadC V. cholerae* grown overnight in anaerobic LB culture, pH reduced with malic acid. (k) wild-type (WT) and and Δ*cadC V. cholerae* grown overnight in aerobic bile culture, pH reduced with malic acid.

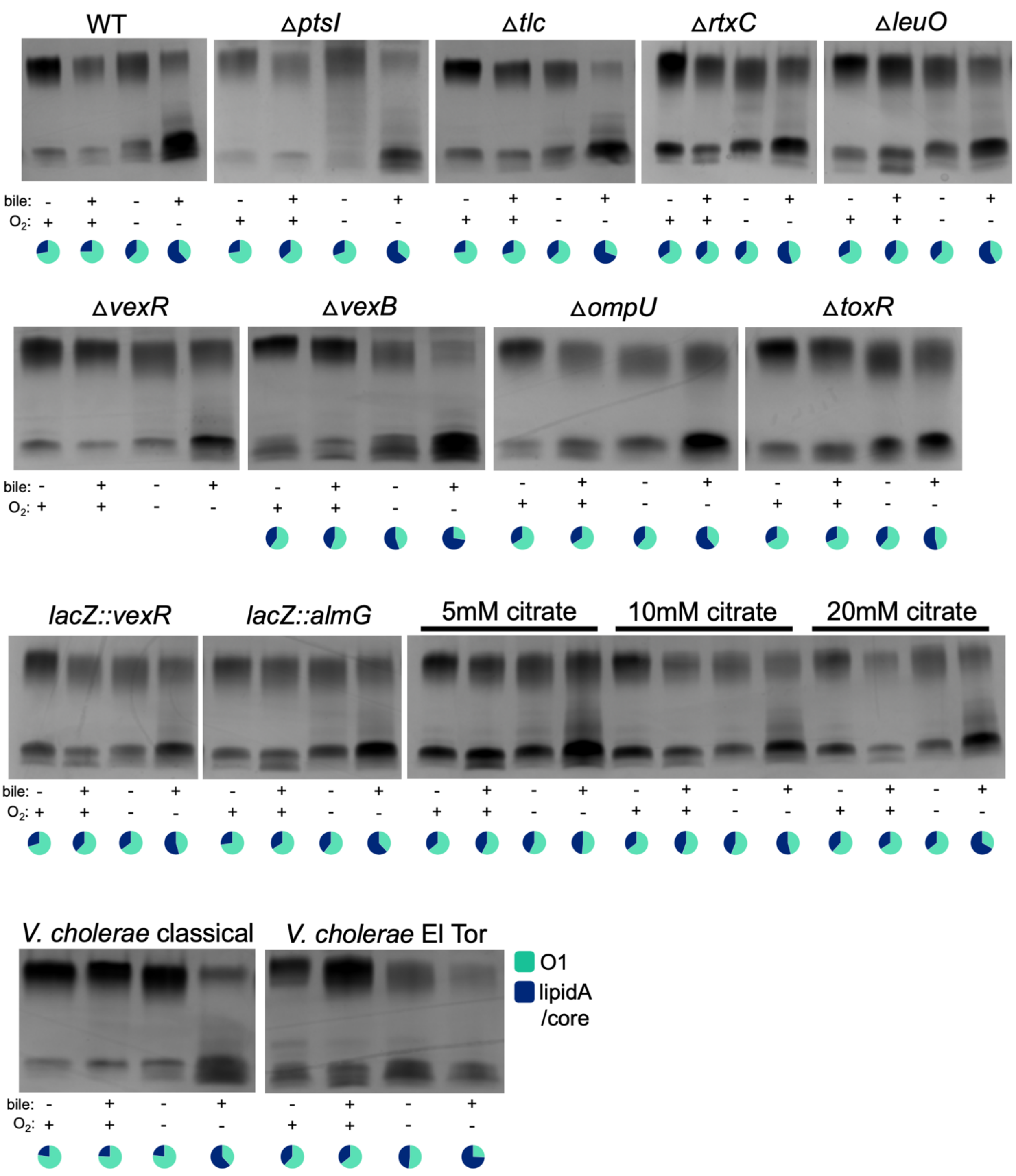

Supplementary Figure S11

Representative silver stain gels of LPS purified from *V. cholerae* with indicated genotype (WT: wild type) grown overnight in LB or LB supplemented with 0.5% bile (bile +/-), aerobically or anaerobically (O_2 +/-). Gene references are provided in Supplementary Table 2. Gel labeled with citrate represents wild-type *V. cholerae* grown in the indicated concentration of sodium citrate (mM). Pie charts underneath gel images represent pixel intensity quantification (n≥2) of the

resulting fraction of intensity from lipid A/core (dark blue) and O1-antigen (light green). Quantification was excluded for experiments where only one replicate was conducted (n=1). "*V. cholerae* classical" is a cholera patient isolate from Egypt (1949). "*V. cholerae* clinical" is a cholera patient isolate from Bangladesh (2011). The strain list in Supplementary Table 1 contains reference and source information for all strains. Quantification method for silver stain gels in this figure differs from the others in the text: this figure only does not include background subtraction in the pixel intensity quantification.

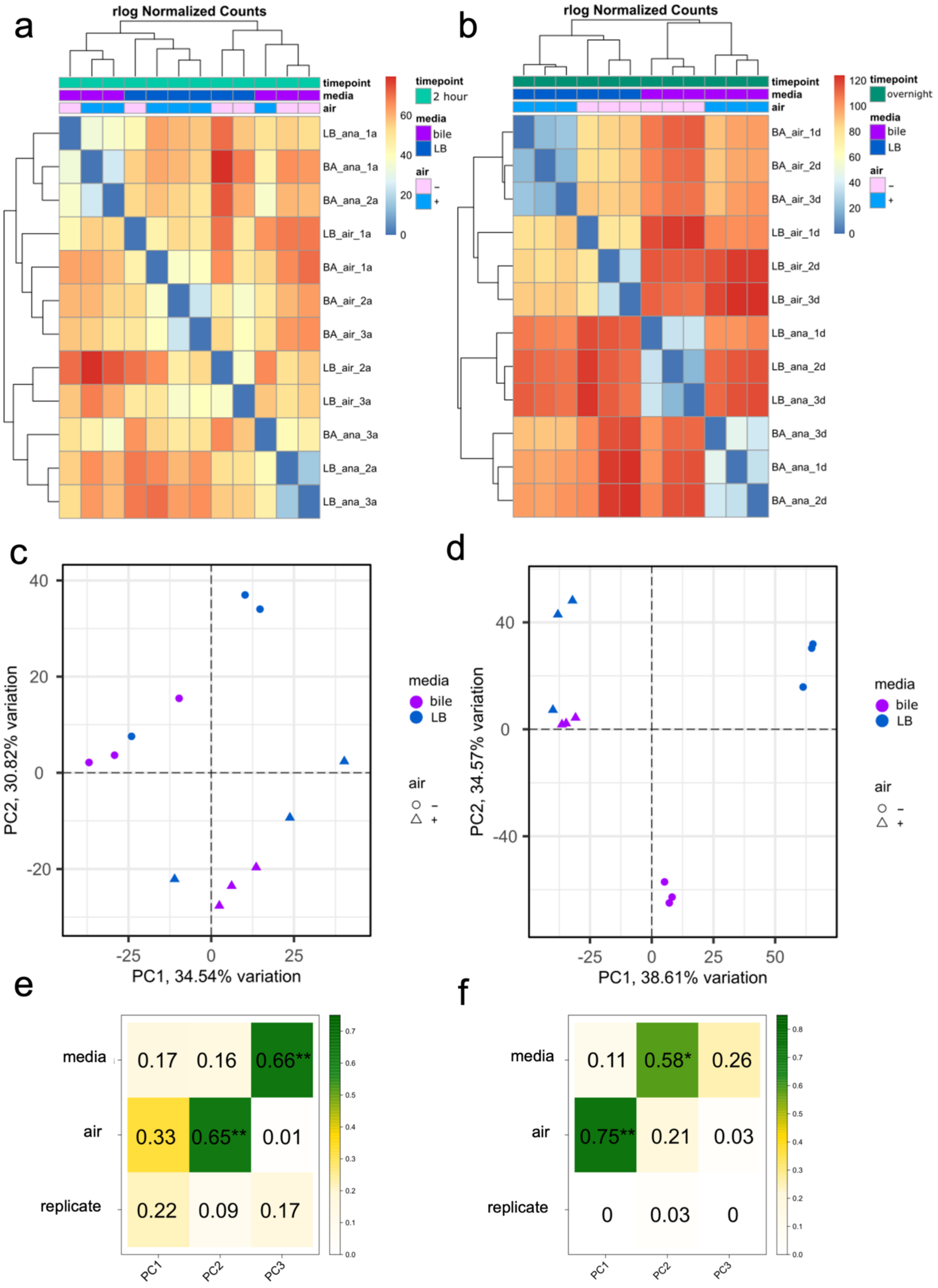
a
rlog Normalized Counts
timepoint
media
air
LB_ana_1a
BA_ana_1a
BA_ana_2a
LB_air_1a
BA_air_1a
BA_air_2a
BA_air_3a
LB_air_2a
LB_air_3a
BA_ana_3a
LB_ana_2a
LB_ana_3a
60
40
20
0
timepoint
2 hour
media
bile
LB
air
-
+
b
rlog Normalized Counts
timepoint
media
air
BA_air_1d
BA_air_2d
BA_air_3d
LB_air_1d
LB_air_2d
LB_air_3d
LB_ana_1d
LB_ana_2d
LB_ana_3d
BA_ana_3d
BA_ana_1d
BA_ana_2d
120
100
80
60
40
20
0
timepoint
overnight
media
bile
LB
air
-
+
c
PC2, 30.82% variation
PC1, 34.54% variation
40
20
0
-20
-25
0
25
media
bile
LB
air
-
+
d
PC2, 34.57% variation
PC1, 38.61% variation
40
0
-40
-25
0
25
50
media
bile
LB
air
-
+
e
media
air
replicate
0.17
0.16
0.66**
0.33
0.65**
0.01
0.22
0.09
0.17
PC1
PC2
PC3
f
media
air
replicate
0.11
0.58*
0.26
0.75**
0.21
0.03
0
0.03
0
PC1
PC2
PC3

Supplementary Figure S12

Quality assurance analysis of the RNA sequencing individual replicates

(**a**) Two-hour and (**b**) overnight timepoint Euclidian distance matrix, representing the similarity of the individual biological replicates based on the regular logarithmic transformation method. Samples are clustered according to distance and the heatmap scale shows the relative distance between clusters, with lower values representing more similar clusters. (**c**) Two-hour and (**d**) overnight timepoint PCA biplots, showing the comparison of principal components 1 and 2 for each timepoint of samples. Samples are colored according to presence (purple) or absence (blue) of bile in the media conditions and shapes are according to the presence (triangle) or absence (circle) of oxygen during culture. Percent variance described by each principal component is plotted on the x and y axis. (**e**) Two-hour and (**f**) overnight timepoint Eigencor plots, showing the impact of media, aerobicity, and replicates on the variance described by each principal component. The plotted values represent the Pearson R^2 coefficient of each treatment across a given principal component. The number of principal components plotted was chosen according to Horn's method in the PCAtools R package.

Supplementary Table 1. Strains used in this study
*Reference numbers refer to reference list in text. References with roman numerals in parenthesis are additional references listed below the tables

Name in text	Strain	Description	Source*
WT	KDS6	*V. cholerae* E7946. O1, El Tor Ogawa, CTX(+), streptomycin resistant	Lab collection
ICP1		ICP1_2006_E ΔCRISPR ΔCas2-3	(I)
ICP2			Lab collection
ICP3			Lab collection
Δ*wbeL*	KS201	*V. cholerae* E7946, *wbeL* open reading frame deleted	24
wbeE-3xFLAG	ZN398	*V. cholerae* E7946, *vc0244 (wbeE)* tagged with a C-terminal 3x-FLAG tag in the native locus. Also contains a kanamycin resistance cassette in the *lacZ* locus	This study
wbeU-3xFLAG	ZN403	*V. cholerae* E7946, *vc0259 (wbeU)* tagged with a C-terminal 3x-FLAG tag in the native locus. Also contains a kanamycin resistance cassette in the *lacZ* locus	This study
Δ*cadC*	ZN350	*V. cholerae* E7946, *cadC* gene replaced with a spectinomycin resistance cassette flanked by FRT sites	This study
Δ*cadA*	ZN356	*V. cholerae* E7946, *cadA* gene replaced with a spectinomycin resistance cassette flanked by FRT sites	This study
Δ*ptsI*	ZN329	*V. cholerae* E7946, *ptsI* gene replaced with a spectinomycin resistance cassette flanked by FRT sites	This study
Δ*tlc*	ZN326	*V. cholerae* E7946, *tlc* genes replaced with a spectinomycin resistance cassette flanked by FRT sites	This study
Δ*rtxC*	ZN341	*V. cholerae* E7946, *rtxC* gene replaced with a spectinomycin resistance cassette flanked by FRT sites	This study
Δ*leuO*	ZN344	*V. cholerae* E7946, *leuO* gene replaced with a spectinomycin resistance cassette flanked by FRT sites	This study
Δ*vexR*	ZN347	*V. cholerae* E7946, *vexR* gene replaced with a spectinomycin resistance cassette flanked by FRT sites	This study
Δ*vexB*	ZN57	*V. cholerae* E7946, *vexB* gene replaced with a spectinomycin resistance cassette flanked by FRT sites	This study

Δ*ompU*	KS799	*V. cholerae* E7946, *ompU* open reading frame deleted	(II)
Δ*toxR*	KS801	*V. cholerae* E7946, *toxR* open reading frame deleted	(III)
lacZ::EV	KS1500	*V. cholerae* E7946, *lacZ* gene replaced with an empty cassette under IPTG-inducible (P_{tac}) and theophylline-inducible (RiboE) promoters and a kanamycin resistance marker	(I)
lacZ::vexR	ZN332	*V. cholerae* E7946, *lacZ* gene replaced with *vexR* under IPTG-inducible (P_{tac}) and theophylline-inducible (RiboE) promoters and a kanamycin resistance marker	This study
lacZ::almG	ZN335	*V. cholerae* E7946, *lacZ* gene replaced with *almG* under IPTG-inducible (P_{tac}) and theophylline-inducible (RiboE) promoters and a kanamycin resistance marker	This study
lacZ::cadC	ZN338	*V. cholerae* E7946, *lacZ* gene replaced with *cadC* under IPTG-inducible (P_{tac}) and theophylline-inducible (RiboE) promoters and a kanamycin resistance marker	This study
V. cholerae classical	KS808	*V. cholerae* clinical patient isolate from Egypt, 1949	(IV)
V. cholerae El Tor	KDS1	*V. cholerae* clinical patient isolate from Bangladesh, 2011	(V)

Supplementary Table 2. Gene names references in text
*Reference numbers refer to reference list in text. References with roman numerals in parenthesis are additional references listed below the tables

name (text)	VCID	locus tag	annotation	description	ref*
vexA	VC0165	CSW01_RS00860	hypothetical protein	RND bile efflux component	34
vexB	VC0164	CSW01_RS00855	multidrug resistance protein	RND bile efflux component	34
vexC	VC1756	CSW01_RS08835	periplasmic linker protein	RND bile efflux component	34
vexD	VC1757	CSW01_RS08840	AcrB/AcrD/AcrF family transporter	RND bile efflux component	34
ompU	VC0633	CSW01_RS03335	porin	outer membrane porin involved in bile resistance	30

tcpI	VC0825	CSW01_ RS04260	toxin co-regulated pilus biosynthesis protein I	TCP methyl-accepting chemoreceptor	37
tcpP	VC0826	CSW01_ RS04265	toxin co-regulated pilus biosynthesis protein P	TCP putative regulatory protein	37
tcpH	VC0827	CSW01_ RS04270	toxin co-regulated pilus biosynthesis protein H		37
tcpA	VC0828	CSW01_ RS04275	toxin co-regulated pilin	TCP major subunit	37
tcpB	VC0829	CSW01_ RS04280	toxin co-regulated pilus biosynthesis protein B	TCP minor subunit	37
tcpQ	VC0830	CSW01_ RS04285	toxin co-regulated pilus biosynthesis protein Q		37
tcpC	VC0831	CSW01_ RS04290	toxin co-regulated pilus biosynthesis outer membrane protein C	TCP outer membrane lipoprotein	37
tcpR	VC0832	CSW01_ RS04295	toxin co-regulated pilus biosynthesis protein R		37
tcpD	VC0833	CSW01_ RS04300	toxin co-regulated pilus biosynthesis protein D		37
tcpS	VC0834	CSW01_ RS04305	toxin co-regulated pilus biosynthesis protein S		37
tcpT	VC0835	CSW01_ RS04310	toxin co-regulated pilus biosynthesis protein T	TCP membrane-associated ATPase	37
tcpE	VC0836	CSW01_ RS04315	toxin co-regulated pilus biosynthesis protein E		37
tcpF	VC0837	CSW01_ RS04320	toxin co-regulated pilus biosynthesis protein F	TCP putative outer membrane channel	37
toxT	VC0838	CSW01_ RS04325	TCP pilus virulence regulatory protein	regulatory protein	36
tcpJ	VC0839	CSW01_ RS04330	leader peptidase TcpJ	signal peptidase	37
acfB	VC0840	CSW01_ RS04335	accessory colonization factor AcfB	accessory colonization factor	39
acfC	VC0841	CSW01_ RS04340	accessory colonization factor AcfC	accessory colonization factor	40
acfA	VC0844	CSW01_ RS04355	accessory colonization factor AcfA	accessory colonization factor	38
acfD	VC0845	CSW01_ RS04360	accessory colonization factor AcfD	accessory colonization factor	(VI)
toxS	VC0983	CSW01_ RS05035	regulatory protein ToxS		36
toxR	VC0984	CSW01_ RS05040	cholera toxin transcriptional activator		36
aphB	VC1049	CSW01_ RS05355	LysR family transcriptional regulator	accessory virulence factor	85

aphA	VC1050	CSW01_ RS05360	response regulator	accessory virulence factor	86
rstC	VC1452	CSW01_ RS07300	RstC protein		42
rstB	VC1453	CSW01_ RS07305	RstB1 protein		42
rstA	VC1463	CSW01_ RS07310	RstA2 protein		42
rstR	VC1464	CSW01_ RS07315	transcriptional repressor RstR		42
ctxB	VC1456	CSW01_ RS07325	cholera enterotoxin subunit B		41
ctxA	VC1457	CSW01_ RS07330	cholera enterotoxin subunit A		41
ompT	VC1854	CSW01_ RS09310	porin	outer membrane porin	30
leuO	VC2485	CSW01_ RS12615	leucine transcriptional activator		(VII)
almE	VC1577	CSW01_ RS07935	enterobactin synthetase subunit F	lipid A modification amino acid ligase	46
almF	VC1578	CSW01_ RS07940	hypothetical protein	lipid A modification glycine carrier protein	46
almG	VC1579	CSW01_ RS07945	hypothetical protein	lipid A modification glycine transferase	46
vexR	VC0166	CSW01_ RS00865	TetR family transcriptional regulator	bile RND efflux regulator	34
ptsI	VC0965	CSW01_ RS04940	phosphoenolpyruvate-protein phosphotransferase EI		52
tlc	VC1466-70, VC1472-6	CSW01_ RS07380 -405, CSW01_ RS07415 -45		Satellite phage of CTX (2 copies)	(VIII)
cadC	VC0278	CSW01_ RS01450	DNA-binding transcriptional activator CadC	weak acid tolerance system	55
cadB	VC0280	CSW01_ RS01455	lysine/cadaverine antiporter	weak acid tolerance system	55
cadA	VC0281	CSW01_ RS01460	lysine decarboxylase, inducible	weak acid tolerance system	55
rtxA	VC1451	CSW01_ RS07295	RTX toxin RtxA		53
rtxC	VC1450	CSW01_ RS07290	RTX toxin activating protein		53

clcA	VCA0526	CSW01_ RS16970	chloride channel protein		65
makD	VC0880	CSW01_ RS04530	hypothetical protein	motility associated killing factor D	77
makC	VC0881	CSW01_ RS04535	hypothetical protein	motility associated killing factor C	77
makB	VC0882	CSW01_ RS04540	hypothetical protein	motility associated killing factor B	77
makA	VC0884	CSW01_ RS04545	acetyltransferase-like protein	motility associated killing factor A	77
aceA-1	VC0734	CSW01_ RS03835	malate synthase		80
aceA-2	VCA0957	CSW01_ RS19015	malate synthase		80
glmS	VC0487	CSW01_ RS02600	glucosamine--fructose-6-phosphate aminotransferase		(IX)
vprA	VC1320	CSW01_ 06665	DNA-binding response regulator aka carR		47
vprB	VC1319	CSW01_ RS06680	sensor histidine kinase aka carS		47
manC	VC0241	CSW01_ RS01280	mannose-1-phosphate guanylyltransferase	O-biosynthetic protein-perosamine	48
manB	VC0242	CSW01_ RS01285	phosphomannomutase	O-biosynthetic protein-perosamine	48
gmd	VC0243	CSW01_ RS01290	GDP-mannose 4,6-dehydratase	O-biosynthetic protein-perosamine	48
wbeE	VC0244	CSW01_ RS01295	perosamine synthase	O-biosynthetic protein-perosamine	48
wbeG	VC0245	CSW01_ RS01300	RfbG protein	O-biosynthetic protein-other	48
wzm	VC0246	CSW01_ RS01305	lipopolysaccharide/O-antigen transport protein	O-biosynthetic protein-transport	48
wzt	VC0247	CSW01_ RS01310	lipopolysaccharide/O-antigen transport protein	O-biosynthetic protein-transport	48
wbeK	VC0248	CSW01_ RS01315	acyl carrier protein	O-biosynthetic protein-tetronate	48
wbeL	VC0249	CSW01_ RS01320	RfbL protein	O-biosynthetic protein-tetronate	48
wbeM	VC0250	CSW01_ RS01325	iron-containing alcohol dehydrogenase	O-biosynthetic protein-tetronate	48
wbeN	VC0251	CSW01_ RS01330	acyl protein synthase/acyl-CoA reductase RfbN	O-biosynthetic protein-tetronate	48

wbeO	VC0252	CSW01_RS01335	acetyltransferase	O-biosynthetic protein-tetronate	48
wbeP	VC0253	CSW01_RS01340	unknown	O-biosynthetic protein-tetronate	48
IS1358d1	VCA0493	CSW01_RS01345	IS1004 transposase		48
wbeT	VC0258	CSW01_RS01355	RfbT-like protein	O-biosynthetic protein-tetronate	48
wbeV	VC0259	CSW01_RS01360	lipopolysaccharide biosynthesis protein RfbV	O-biosynthetic protein-other	48
wbeU	VC0260	CSW01_RS01365	mannosyltransferase	O-biosynthetic protein-other	48
galE	VC0262	CSW01_RS01370	UDP-glucose 4-epimerase	O-biosynthetic protein-other	48
wbeW	VC0263	CSW01_RS01375	galactosyl-transferase	O-biosynthetic protein-other	48
wavA	VC0223	CSW01_RS01195	ADP-heptose--LPS heptosyltransferase II	core-polysaccharide biosynthesis	48
wavB	VC0224	CSW01_RS01200	lipopolysaccharide biosynthesis glycosyltransferase	core-polysaccharide biosynthesis	48
waaC	VC0225	CSW01_RS01205	lipopolysaccharide biosynthesis protein	core-polysaccharide biosynthesis	48
wavC	VC0227	CSW01_RS01210	3-deoxy-D-manno-octulosonic acid kinase	core-polysaccharide biosynthesis	48
wavD	VC0228	CSW01_RS01215	hypothetical protein	core-polysaccharide biosynthesis	48
wavE	VC0229	CSW01_RS01220	hypothetical protein	core-polysaccharide biosynthesis	48
wavF	VC0230	CSW01_RS01225	hypothetical protein	core-polysaccharide biosynthesis	48
wavG	VC0231	CSW01_RS01230	hypothetical protein	core-polysaccharide biosynthesis	48
wavH	VC0232	CSW01_RS01235	hypothetical protein	core-polysaccharide biosynthesis	48
waaA	VC0233	CSW01_RS01240	3-deoxy-D-manno-octulosonic acid transferase	core-polysaccharide biosynthesis	48
wavI	VC0234	CSW01_RS01245	hypothetical protein	core-polysaccharide biosynthesis	48
wavJ	VC0235	CSW01_RS01250	lipopolysaccharide biosynthesis protein	core-polysaccharide biosynthesis	48
waaF	VC0236	CSW01_RS01255	ADP-heptose--LPS heptosyltransferase II	core-polysaccharide biosynthesis	48

waaL	VC0237	CSW01_ RS01260	hypothetical protein	core-polysaccharide biosynthesis	48
wavK	VC0238	CSW01_ RS01265	hexapaptide repeat-containing transferase	core-polysaccharide biosynthesis	48
wavL	VC0239	CSW01_ RS01270	hypothetical protein	core-polysaccharide biosynthesis	48
gmhD	VC0240	CSW01_ RS01275	ADP-L-glycero-D-mannoheptose-6-epimerase	core-polysaccharide biosynthesis	48
eptA	VCA1102	CSW01_ RS19695	hypothetical protein	lipid A biosynthesis	(X)
gmhA	VC2230	CSW01_ RS11395	phosphoheptose isomerase	lipid A biosynthesis	(X)
gmhB	VC0908	CSW01_ RS04660	D,D-heptose 1,7-bisphosphate phosphatase	lipid A biosynthesis	(X)
kdsA	VC2175	CSW01_ RS10855	2-dehydro-3-deoxyphosphooctonate aldolase	lipid A biosynthesis	(X)
kdsB	VC1875	CSW01_ RS09420	3-deoxy-manno-octulosonate cytidylyltransferase	lipid A biosynthesis	(X)
lpxA	VC2248	CSW01_ RS11480	acyl-(acyl-carrier-protein)--UDP-N-acetylglucosamine O-acyltransferase	lipid A biosynthesis	(X)
lpxB	VC2247	CSW01_ RS11475	lipid-A-disaccharide synthase	lipid A biosynthesis	(X)
lpxC	VC2396	CSW01_ RS12170	UDP-3-O-[3-hydroxymyristoyl] N-acetylglucosamine deacetylase	lipid A biosynthesis	(X)
lpxD	VC2250	CSW01_ RS11490	UDP-3-O-[3-hydroxymyristoyl] glucosamine N-acyltransferase	lipid A biosynthesis	(X)
lpxH	VC1850	CSW01_ RS09290	UDP-2,3-diacylglucosamine hydrolase	lipid A biosynthesis	(X)
lpxK	VC1877	CSW01_ RS09430	tetraacyldisaccharide 4'-kinase	lipid A biosynthesis	(X)
lpxL	VC0213	CSW01_ RS01140	lipid A biosynthesis lauroyl acyltransferase	lipid A biosynthesis	(X)
lpxM	VC0212	CSW01_ RS01135	lipid A biosynthesis (KDO)2-(lauroyl)-lipid IVA acyltransferase	lipid A biosynthesis	(X)

lpxR	VC1867	CSW01_ RS09380	hypothetical protein	lipid A biosynthesis	(X)

Additional references for supplementary tables:
(I) McKitterick AC, Seed KD. Anti-phage islands force their target phage to directly mediate island excision and spread. *Nat Commun*. 2018;9(1):2348. doi:10.1038/s41467-018-04786-5
(II) Schild S, Nelson EJ, Camilli A. Immunization with *Vibrio cholerae* Outer Membrane Vesicles Induces Protective Immunity in Mice. *Infect Immun*. 2008;76(10):4554-4563. doi:10.1128/IAI.00532-08
(III) Anthouard R, DiRita VJ. Small-Molecule Inhibitors of *toxT* Expression in Vibrio cholerae. Taylor R, ed. *mBio*. 2013;4(4):e00403-13. doi:10.1128/mBio.00403-13
(IV) Mutreja A, Kim DW, Thomson NR, et al. Evidence for several waves of global transmission in the seventh cholera pandemic. *Nature*. 2011;477(7365):462-465. doi:10.1038/nature10392
(V) Seed KD, Lazinski DW, Calderwood SB, Camilli A. A bacteriophage encodes its own CRISPR/Cas adaptive response to evade host innate immunity. *Nature*. 2013;494(7438):489-491. doi:10.1038/nature11927
(VI) Peterson KM, Mekalanos JJ. Characterization of the Vibrio cholerae ToxR Regulon: Identification of Novel Genes Involved in Intestinal Colonization. *Infect Immun*. 1988;56:8.
(VII) Bina XR, Taylor DL, Vikram A, Ante VM, Bina JE. Vibrio cholerae ToxR Downregulates Virulence Factor Production in Response to Cyclo(Phe-Pro). DiRita V, Collier RJ, eds. *mBio*. 2013;4(5):e00366-13. doi:10.1128/mBio.00366-13
(VIII) Hassan F, Kamruzzaman M, Mekalanos JJ, Faruque SM. Satellite phage TLCϕ enables toxigenic conversion by CTX phage through dif site alteration. *Nature*. 2010;467(7318):982-985. doi:10.1038/nature09469
(IX) Ghosh S, Blumenthal HJ, Davidson E, Roseman S. Glucosamine Metabolism. *Journal of Biological Chemistry*. 1960;235(5):1265-1273. doi:10.1016/S0021-9258(18)69397-4
(X) Klein G, Raina S. Regulated Assembly of LPS, Its Structural Alterations and Cellular Response to LPS Defects. *IJMS*. 2019;20(2):356. doi:10.3390/ijms20020356
